# High-Throughput Field Phenotyping Using Unmanned Aerial Vehicles (UAVs) for Rapid Estimation of Photosynthetic Traits

**DOI:** 10.1016/j.plaphe.2025.100045

**Published:** 2025-04-26

**Authors:** Jingshan Lu, Qimo Qi, Gangjun Zheng, Jan U.H. Eitel, Qiuyan Zhang, Jiuyuan Zhang, Sumei Chen, Fei Zhang, Weimin Fang, Zhiyong Guan, Fadi Chen

**Affiliations:** a State Key Laboratory of Crop Genetics & Germplasm Enhancement and Utilization, Key Laboratory of Landscaping, Ministry of Agriculture and Rural Affairs, Key Laboratory of Biology of Ornamental Plants in East China, National Forestry and Grassland Administration, College of Horticulture, Nanjing Agricultural University, Nanjing, Jiangsu, 210095, China; b Department of Natural Resources and Society, College of Natural Resources, University of Idaho (UI), 875 Perimeter Drive, Moscow, ID, 83843, USA; c McCall Outdoor Science School, College of Natural Resources, University of Idaho, 1800 University Lane, McCall, ID, 83638, USA

**Keywords:** Unmanned aerial vehicle (UAV), Photosynthetic traits, Variable selection, Machine learning, Photosynthesis mapping

## Abstract

Efficient measurement of photosynthetic traits, such as the maximum carboxylation rate of Rubisco (Vcmax) and electron transport rate (Jmax), is essential for advancing research and breeding aimed at enhancing crop productivity. Traditional methods are time-intensive, which limits their scalability. Remote sensing presents an opportunity for estimating these traits; however, it often lacks an affordable platform for effective spatial mapping, a critical aspect of phenotyping. This study explored the use of unmanned aerial vehicle (UAV) multispectral data to estimate and spatially map photosynthetic traits in tea chrysanthemums during the branching and budding stages under an open canopy. Over six field experiments across varieties conducted in 2022–2023, we captured canopy reflectance using UAV-mounted multispectral sensors, calculated spectral indices, and measured the photosynthetic traits of the upper leaves using a portable photosynthesis system. The results indicated that certain indices, particularly those incorporating green and red-edge bands, effectively estimated photosynthetic traits, with the simplified canopy chlorophyll content index (SCCCI) yielding the most accurate Vcmax estimates (R^2^ ​= ​0.52) and the chlorophyll vegetation index (CVI) providing the best estimates for Jmax (R^2^ ​= ​0.38). The integration of variable selection with partial least squares regression (PLSR) modeling further enhanced the precision of the model (Vcmax: R^2^ ​= ​0.70; Jmax: R^2^ ​= ​0.63). Our findings demonstrate that UAV-acquired multispectral data can effectively map photosynthetic traits with high spatial resolution, establishing it as a valuable tool for rapid phenotyping and spatial assessment of photosynthetic capacity in crop fields.

## 1 Introduction

Photosynthesis serves as the foundation for plant growth, development, and dry matter accumulation [1]. Enhancing photosynthetic efficiency is widely recognized as a crucial factor for increasing crop yields [2], making the development of high-light-efficiency crop varieties a key priority for breeders. Leaf photosynthetic capacity is primarily determined by two key traits: maximum carboxylation rate (Vcmax) and maximum electron transport rate (Jmax). Vcmax represents the maximum rate at which Ribulose−1,5-Bi-sphosphate (RuBP) is carboxylated and directly influences CO_2_ fixation [3]. Jmax indicates the maximum electron transport rate, which constrains the availability of ATP and NADPH during both the carboxylation process and the regeneration of RuBP within the Calvin-Benson cycle [4]. Traditionally, these photosynthetic traits are measured using a gas exchange system (LI-6400 or LI-6800; Li-Cor, Lincoln, NE, USA) to obtain photosynthesis-intercellular carbon dioxide (CO_2_) response curves (A-Ci curves) [3]. Vcmax and Jmax values are then calculated by fitting the A-Ci curve using the Farquhar–von Caemmerer–Berry (FvCB) model [5]. However, measuring the A-Ci curves is time-consuming and labor-intensive, requiring approximately 30 ​min per sample with LI-6400 [6] and 15 ​min with LI-6800 [7]. Therefore, there is a pressing need for a method that can quickly and accurately estimate crop photosynthetic traits in real-time, which is essential for advancing breeding programs.

Remote sensing (RS) techniques have been successfully applied to monitor vegetation photosynthetic traits, including *in situ* leaf hyperspectral reflectance [[8], [9], [10], [11], [12], [13], [14], [15], [16], [17], [18], [19], [20], [21]], proximal hyperspectral imagery [[22], [23], [24]], airborne imagery [[25], [26], [27]], and satellite imagery [28,29]. For example, Buchaillot et al. [8] used *in situ* leaf hyperspectral reflectance to estimate the Vcmax and Jmax of soybeans (*Glycine max*. L), and peanut (*Arachis hypogea* L.). Similarly, Zhi et al. [24] demonstrated that sorghum (*Sorghum bicolo*r (L.) Moench) Vcmax and Jmax could be estimated using proximal hyperspectral imagery. Serbin et al. [26] employed airborne hyperspectral imagery to estimate vegetation Vcmax. Chen et al. [28] utilized TROPical Ozone Mission (TROPOMI) satellite imagery to estimate global vegetation Vcmax. While the studies mentioned above demonstrate the effectiveness of *in situ* and proximal hyperspectral remote sensing for detecting variations in photosynthetic traits, these methods are costly and unsuitable for mapping these traits across different varieties across farm fields [23]. Airborne remote sensing offers a viable alternative, but requires aircraft scheduling and is still expensive. Finally, satellite imagery can be used to monitor photosynthetic traits remotely; however, it is limited by weather conditions and typically has a low spatial resolution [30]. Moreover, the spatial resolution of airborne and satellite imagery is too low to be suitable for collecting photosynthetic trait data in crop phenotyping and breeding studies that require a spatial resolution of no less than 3 ​cm. The high spatial resolution is particularly important during the early stages of crop growth to distinguish vegetation spectral information from that of the soil background.

Unmanned aerial vehicles (UAVs) are increasingly favored for estimating crop phenotypic parameters owing to their cost-effectiveness, fine spatial resolution (e.g., centimeters), and flexible image capture capabilities [[31], [32], [33], [34], [35], [36], [37], [38]]. Several studies have employed UAVs to estimate leaf net photosynthesis of crops [39,40]. For example, Zhang et al. [40] successfully estimated leaf maximum net photosynthesis in rice (*Oryza sativa* L.) using the modified structure insensitive pigment index (SIPI_m_ (R_720_-R_550_)/(R_800_-R_680_)) derived from UAV multispectral imagery. However, to date, we are aware of no research that has applied UAV remote sensing imagery to estimate crop Vcmax and Jmax values. Previous studies have suggested that the mechanism for estimating photosynthetic traits via hyperspectral RS is primarily driven by chlorophyll *a and b* contents (Cab) [29] or leaf N content per unit area (Narea) [9]. This correlation highlights pigment-related spectral features, particularly in the 400–800 ​nm range, with the red-edge region (680–730 ​nm) being most strongly associated with photosynthetic traits [13,22,23]. Most commercially available UAV multispectral cameras capture red, green, blue, red-edge, and near-infrared bands [41]. The spectral indices derived from these bands hold promise for estimating Vcmax and Jmax, offering a new avenue for high-throughput screening of photosynthetic traits in crops.

Machine learning algorithms, in addition to spectral indices, have been widely applied to estimate vegetation photosynthetic traits because of their ability to efficiently process large-scale data, capture complex nonlinear relationships, and enhance the accuracy of agricultural phenotypic parameter inversion [8,10,42]. Among these algorithms, partial least squares regression (PLSR) is widely regarded as the most popular method for estimating vegetation photosynthetic traits. PLSR is highly effective in modeling the relationship between numerous predictor variables (e.g., spectral bands) and target responses, efficiently handling multicollinearity while providing a robust model for high-dimensional data [13,15,22,23]. However, hyperspectral data often contain redundant or highly correlated information, which can increase model complexity and reduce interpretability. To address this, variable selection methods like the least absolute shrinkage and selection operator (LASSO) have been introduced. Unlike other variable selection techniques, LASSO applies L1 regularization to shrink the coefficients of less important variables to zero, making it particularly effective at identifying the most relevant spectral features. This process not only reduces model overfitting but also enhances interpretability [43]. As a result, LASSO has gained recognition as a powerful tool for variable selection in precision agriculture [44,45]. For example, Song et al. [46] effectively estimated the leaf photosynthetic traits of tree species (Fagus crenata Blume) by combining LASSO-based hyperspectral variable selection with machine learning algorithms. In summary, PLSR is well-suited for high-dimensional datasets with multicollinearity, capturing the overall structure of the data. In contrast, LASSO excels at simplifying models by selecting a smaller subset of important variables, thereby improving model interpretability. Integrating both methods—using LASSO for variable selection and PLSR for modeling—combines the strengths of both approaches, resulting in more efficient and interpretable models for estimating vegetation photosynthetic traits.

The overall objective of this research was to establish a model for estimating the photosynthetic traits of tea chrysanthemums (*Chrysanthemum morifolium* Ramat.) using UAV-based multispectral imagery. To achieve this, our specific objectives were: (1) to investigate the relationship between spectral indices derived from UAV multispectral imagery and chrysanthemum photosynthetic traits, (2) to develop a photosynthetic trait estimation model by coupling machine learning with spectral feature selection, and (3) to accurately map the photosynthetic traits of multiple chrysanthemum varieties.

## 2 Materials and methods

### 2.1 Experimental design

**Fig. 1:**
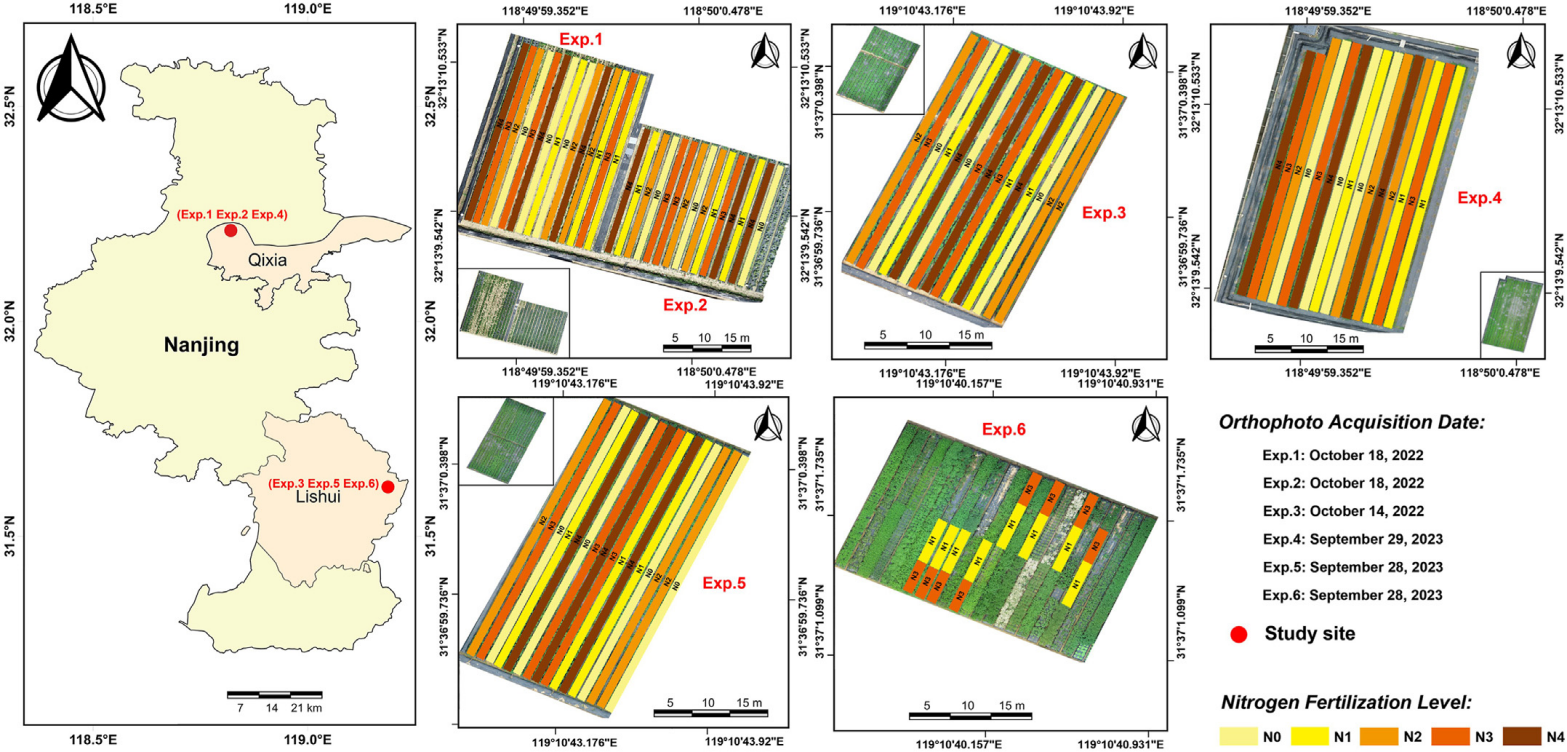
Geographic distribution and experimental design details of the six field experiments. Nitrogen fertilization levels include: 0 ​kg/hm^2^ (N0), 130 ​kg/hm^2^ (N1), 260 ​kg/hm^2^ (N2), 390 ​kg/hm^2^ (N3) and 520 ​kg/hm^2^ (N4). Experiments 1–5 were completely randomized block design with three replicates, and experiment 6 was a multi-variety trial. The plot sizes and planting densities for each experiment were as follows: Exp. 1: 1.1 ​m ​× ​32 ​m (35.2 ​m^2^) with 105–110 plants per plot; Exp. 2: 1.3 ​m ​× ​27 ​m (35.1 ​m^2^) with 180–185 plants per plot; Exp. 3: 1.1 ​m ​× ​31 ​m (34.1 ​m^2^) with 103–108 plants per plot; Exp. 4: 1.1 ​m ​× ​42 ​m (46.2 ​m^2^) with 140–145 plants per plot; and Exp. 5: 1.1 ​m ​× ​40 ​m (44.0 ​m^2^) with 145–150 plants per plot; Exp. 6: 1.3 ​m ​× ​6 ​m (7.8 ​m^2^) with 40–45 plants per plot.

This study was carried out over two years (2022–2023) at two experimental sites in Nanjing, Jiangsu Province, China: the Baguazhou Experimental Base (Qixia District, 118°83′E, 32°21′N) and the Baima Teaching and Research Base (Lishui District, 119°18′E, 31°61′N). The average annual temperature, precipitation, sunshine duration, and frost-free period at Baguazhou were 16.2 ​°C, 1000 ​mm, 2138 ​h, and 230 ​d, respectively, while Baima exhibited slightly different values of 15.4 ​°C, 1035 ​mm, 2074 ​h, and 210 ​d. Soil characteristics varied between sites. Baguazhou had an organic matter content of 7.45 ​g/kg, with available nitrogen, potassium, and phosphorus at 39.98 ​mg/kg, 101.09 ​mg/kg, and 21.88 ​mg/kg, respectively. In contrast, Baima had higher nutrient levels, with organic matter at 14.04 ​g/kg, nitrogen at 71.11 ​mg/kg, potassium at 148.04 ​mg/kg, and phosphorus at 49.70 ​mg/kg. The study included six field experiments conducted over two years (Fig. 1), evaluating nine tea chrysanthemum varieties under five nitrogen gradient treatments (Exp. 6 included two nitrogen levels). Seedlings were initially cultivated in a greenhouse before being transplanted into field plots arranged in a randomized complete block design with three replicates per treatment. Phosphorus and potassium fertilizers (120 ​kg/ha and 259 ​kg/ha, respectively) were evenly applied and incorporated using a rotary tiller. Standard agronomic practices were maintained throughout the study. Detailed experimental information is provided in Table S1 and S2.

### 2.2 Data collection

The data collection in this study consisted of two main parts: (1) the acquisition of UAV-based canopy remote sensing images of tea chrysanthemums and (2) the acquisition of photosynthesis CO_2_ (A-Ci) response curves. The growth stages were selected based on their critical importance in canopy development and photosynthetic acclimation processes. Specifically, the middle/late branching stages correspond to rapid leaf area expansion, while the initial/middle budding stages represent the transition from vegetative to reproductive growth with significant changes in source-sink relationships. This multi-stage sampling design allows capturing the dynamic trajectories of both structural and functional traits across developmental continuum. The specific data collection times are listed in Table S3.

### 2.3 UAV imagery collection

To collect UAV imagery, we used a DJI Inspire 2 quadcopter (DJI, Shenzhen, China), featuring a maximum flight time of 27 ​min, a signal range of up to 7 ​km, and a maximum takeoff weight of 4250 ​g (Fig. S2a). With the downward vision system activated, the GPS hovering accuracy is ±0.1 ​m vertically and ±0.3 ​m horizontally. Additional UAV parameters are listed in Table S4. The UAV was equipped with a RedEdge-MX multispectral camera (MicaSense, Seattle, USA), which is capable of capturing five spectral bands: blue, green, red, red-edge, and near-infrared. The camera specifications include a sensor size of 4.8 ​mm ​× ​3.6 ​mm, a focal length of 5.4 ​mm, a horizontal field of view of 47.2°, a vertical field of view of 35.4°, a pixel size of 3.75 ​μm, and a resolution of 1280 ​× ​960. The central wavelengths and bandwidths of the multispectral camera are outlined in Table S5. UAV imaging was conducted during the branching and budding stages of tea chrysanthemums under an open canopy on clear and cloudless days, between 11:00 and 13:00. The UAV followed a predetermined flight path at a speed of 2 ​m/s with a side overlap rate of 70 ​% and a flight path overlap rate of 80 ​%. The flight altitude was maintained at 30 ​m to achieve a ground spatial resolution of 2 ​cm. The calibration reference panel (CRP) used in this study was a homogeneous scattering panel with an approximately 50 ​% reflectance ratio across the five spectral bands (Fig. S2b). Each panel was equipped with a barcode to enable the automatic application of the CRP reflectance data during image processing. To ensure accurate reflectance calibration, the CRP was imaged with the multispectral camera system before takeoff and after landing. During each flight, images were captured at regular intervals, generating remote sensing data across the blue, green, red, red-edge, and near-infrared bands for the experimental area.

### 2.4 Photosynthetic traits collection

**Fig. 2:**
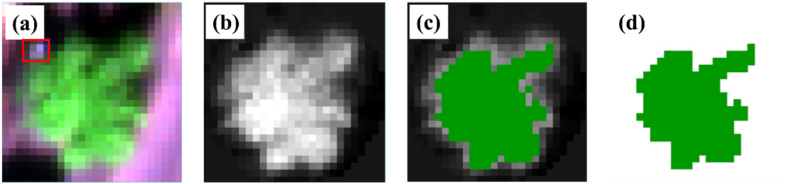
True color image of a plant that was sampled (a), ExG image of the plant (b), ExG image of the plant with a threshold greater than 0.07 (c), ROI of removing soil background and leaf shadows (d). The red frame (a) was a white label used for UAV positioning during A-Ci response curve testing of plants.

Prior to measuring the photosynthetic traits, 20 tea chrysanthemum plants were randomly selected from each plot to assess their plant height and canopy diameter. Based on the average values obtained, one representative plant was chosen and marked with a white label (Fig. 2a) for photosynthetic trait measurements. This plant was consistently used for all subsequent photosynthetic trait measurements at each sampling stage. To collect representative plant leaves for photosynthetic trait analysis, we initially measured the relative chlorophyll content of the top 10 leaves on each plant using a soil and plant analyzer development device (SPAD-502, Minolta Camera Co., Osaka, Japan). Based on these measurements, 1–2 leaves with SPAD values closest to the mean were selected for rapid photosynthetic CO_2_ (A-Ci) response curves measurements using the Li-6800 portable photosynthesis system (Li-Cor BioScience, Lincoln, NE, USA) (Fig. S3). Prior to gas exchange measurements, the leaves were exposed to a light intensity of 1500 ​μmol ​m^−2^ ​s^−1^ at a temperature of 25–30 ​°C for approximately 5 ​min to stabilize. Once acclimated to the instrument's environment, the measurement program was initiated. The instrument increased CO_2_ concentrations from 30 ​μmol ​mol^−1^ to 1830 ​μmol ​mol^−1^ at a rate of 200 ​μmol ​mol^−1^ per minute, recording data every 5 ​s. At the end of the program, the net photosynthetic rates were recorded across 108 CO_2_ concentration levels. Following the measurements, A-Ci response curves were fitted using the R package ‘Plantecophys’ [47] in the R language environment (R Core Team, 2022). Using the FvCB photosynthesis model [3], Vcmax and Jmax values were corrected to 25 ​°C. Photosynthetic trait values for each plant were calculated by averaging the values from the top leaves and detailed information from the different experiments, as shown in Table S6.

It is noteworthy that the higher-than-usual temperatures during the rapid growth phase and the data collection period in 2022 affected the experimental plants. As a result, the leaves required longer stabilization times to acclimate to the photosynthetic instrument environment, with some leaves experiencing stress conditions. Consequently, fewer valid data points were collected. Additionally, the time-intensive process of measuring the A-Ci curves made it infeasible to measure 30 A-Ci curves across 15 plots in a single day. Thus, in the 2023 trial with ‘Fubai chrysanthemum’, only photosynthetic data were collected for the N0, N2, and N4 nitrogen treatments.

### 2.5 UAV data processing

#### 2.5.1 Multispectral remote sensing image processing

Upon completing the aerial operations, the captured images were imported into Pix4Dmapper 4.5.6 (Pix4D SA, Lausanne, Switzerland). Radiometric correction was applied using a CRP, and partial images from each band were stitched together to generate a complete orthorectified reflectance image of the experimental area (Fig. S4a–e). The orthophotos from the five bands were subsequently imported into ENVI (Exelis Visual Information Solutions, Boulder, Colorado, USA) to merge the bands into a single composite image (Fig. S4f). Using the band-fused image, the plant corresponding to the measured A-Ci response curves marked with a white label was delineated using the region of interest (ROI) tool in ENVI (Fig. 2a). To minimize the influence of soil background and leaf shadows on canopy reflectance, the excess green index (ExG) was calculated using the band math tool (Fig. 2b). The threshold range was manually adjusted, and an ExG value above 0.07 was determined as optimal for suppressing background interference effectively (Fig. 2c). This procedure generated an ROI that excluded the soil background and leaf shadows (Fig. 2d). Finally, the average canopy reflectance values of the five bands were extracted from the band-fused image by using the refined ROI.

#### 2.5.2 Published spectral indices

In this study, reflectance data from five bands acquired via UAV imagery were used to compute 63 spectral indices associated with vegetation photosynthetic traits, N, and Cab, to assess their effectiveness in estimating the Vcmax and Jmax of tea chrysanthemum. Further details are provided in Table S7.

#### 2.5.3 Variable selection

To reduce the complexity of the photosynthetic trait estimation model, this study employed the least absolute shrinkage and selection operator (LASSO) algorithm for variable selection among 63 spectral indices. LASSO, first introduced by Tibshirani [43], is a biased estimator that handles multi-collinearity data. The basic LASSO model is a linear regression framework that uses photosynthetic traits as dependent variables and spectral indices as independent variables. It constructs a penalty function to produce a more refined model by shrinking the coefficients of the insignificant variables (spectral indices) to zero, thereby achieving variable selection and model simplification. LASSO regression avoids overfitting by adjusting model complexity, controlled by the parameter λ. A larger λ imposes a greater penalty on the models with many variables, resulting in a model with fewer variables. In this study, the optimal value of λ was determined using 10-fold cross-validation via the cv.glmnet function, with LASSO analysis implemented through the "glmnet" package [48].

#### 2.5.4 Data analysis and photosynthetic traits map

This study analyzed data from six experiments (N ​= ​204) to assess the impact of nitrogen gradient on canopy reflectance and photosynthetic traits in tea chrysanthemums. We also explored the correlations between spectral indices and photosynthetic traits. To evaluate the performance of the spectral indices and machine learning methods for estimating photosynthetic traits, all data from six experiments were randomly split into two sets in a 2:1 ratio: 136 samples were used for calibration, and 68 samples were reserved for validation. Correlations between the spectral indices and photosynthetic traits were visualized and fit with linear regression model using Origin 2022 (OriginLab Corporation, Northampton, Massachusetts, USA). The machine learning algorithm employed was partial least squares regression (PLSR), a linear model implemented through the ‘sklearn’ library in Python 3.11.0. The model hyperparameters were optimized using 10-fold cross-validation. Variable importance in projection (VIP) scores were used to identify the key spectral indices in the PLSR models, with spectral indices considered significant for estimating photosynthetic traits if their VIP scores exceeded 1 [49]. The model performance was assessed using several metrics, including the coefficient of determination (R^2^), absolute correlation coefficient (r), root mean square error (RMSE), bias, and relative root mean square error (rRMSE), all calculated using Python 3.11.0. Additionally, tea chrysanthemum photosynthetic trait maps were generated for a multi-variety experiment (Exp. 6) by integrating RS approaches with the photosynthetic trait model.

## 3 Results

### 3.1 Changes of spectral reflectance and indices under different nitrogen levels

**Fig. 3:**
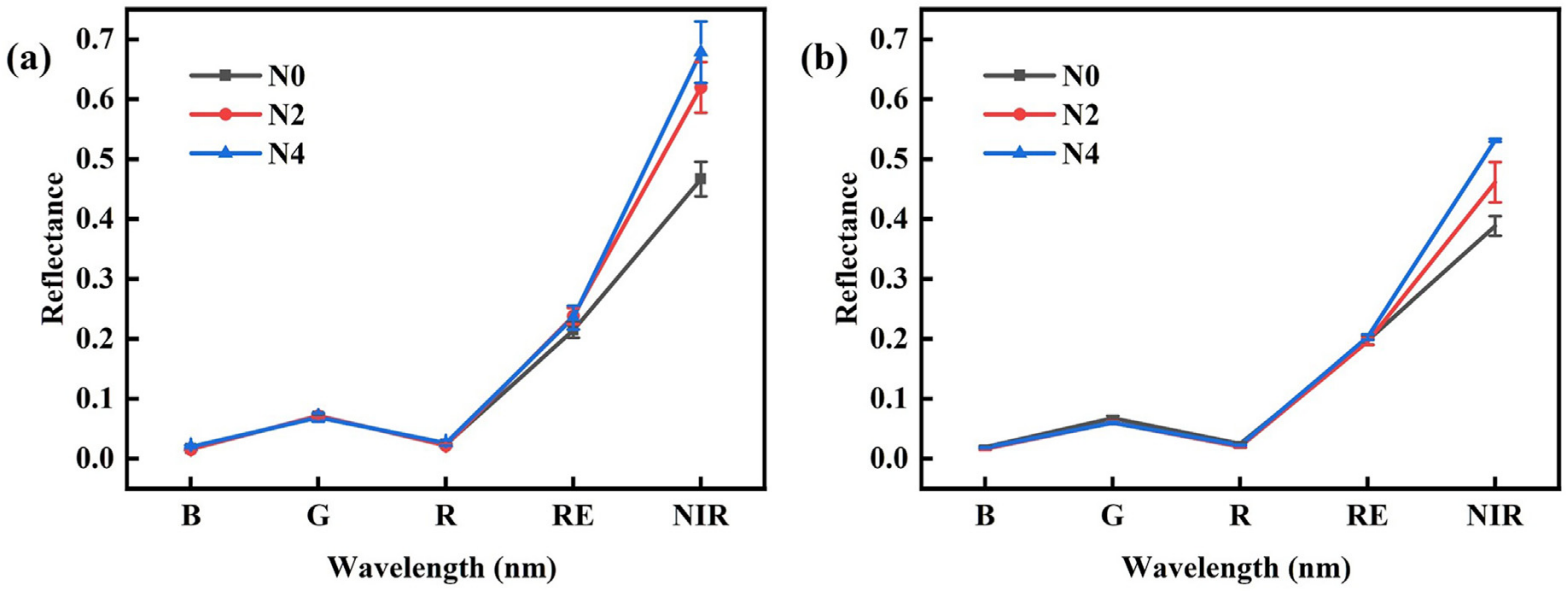
Variation trends of canopy reflectance spectra (with soil and shadow background removed) in tea chrysanthemums under different nitrogen treatments at the initial budding stage: (a) ‘Fubai chrysanthemum’ in Qixia (Exp. 1: 2022.10.12), (b) ‘Fubai chrysanthemum’ in Lishui (Exp. 3: 2022.10.11). The reflectance spectra presented are aggregated values from ROIs collected at the initial budding stage in both experiments. The other varieties and growth stages exhibited similar trends.

**Fig. 4:**
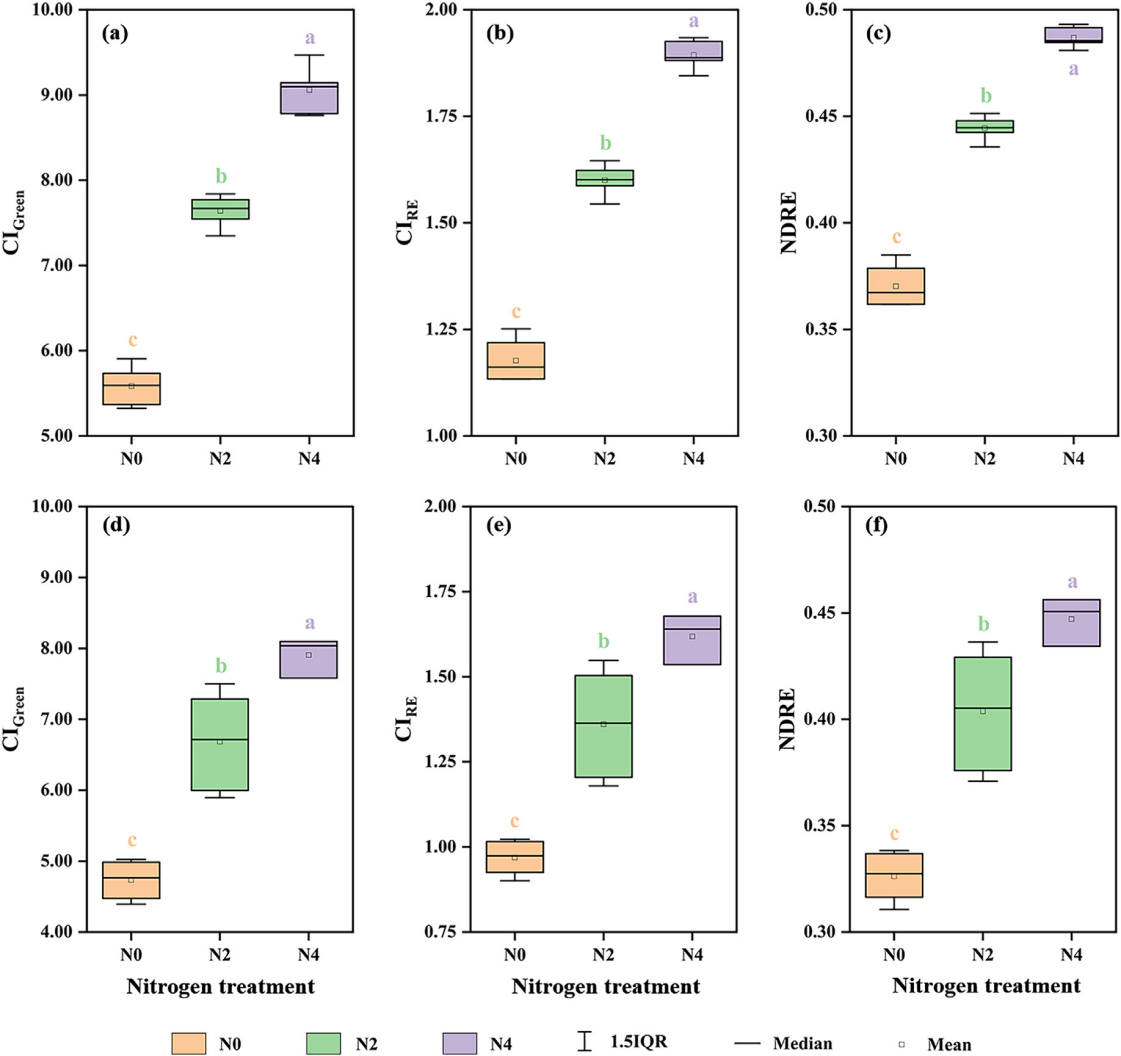
Variation trends of spectral indices for ‘Fubai chrysanthemum’ under different nitrogen treatments in Qixia (Exp. 1: 2022.10.12) (a–c) and Lishui (Exp. 3: 2022.10.11) (d–f). The other varieties and growth stages exhibited similar trends. Different lowercase letters indicate significant differences at the 5 ​% level. IQR was interquartile range.

To visually illustrate the characteristics of multispectral reflectance, we selected 2023 data showing the variety ‘Fubai chrysanthemum’ at the vegetative growth stage under different N levels (Fig. 3). Results showed that reflectance across five bands, extracted from the UAV-mounted multispectral camera, displayed typical vegetation reflection patterns: the NIR band showed the highest reflectance, followed by the RE band, along with a characteristic green peak, red valley, and blue valley. Fig. 4 shows the response variation of previously published spectral indices sensitive to Cab and N (Table S7) under different N levels. The results indicated that the spectral indices CI_Green_, CI_RE_, and NDRE increased with increasing N application levels.

### 3.2 Correlation of tea chrysanthemum photosynthetic traits with published spectral indices

**Fig. 5:**
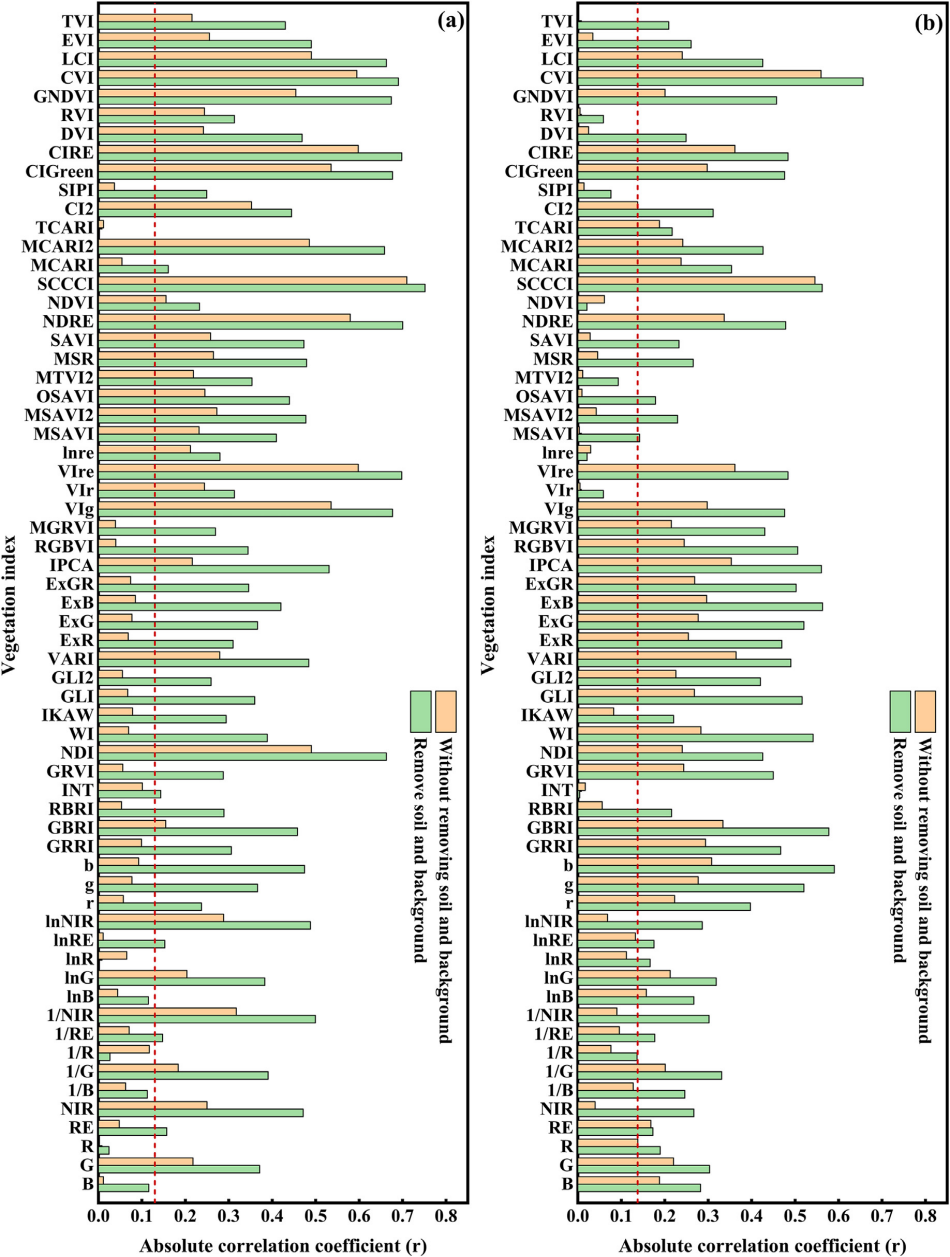
Absolute correlation coefficient (r) between photosynthetic traits and published spectral indices: (a) Vcmax and (b) Jmax. The red dashed line represents a significant correlation at the 0.05 level. The total number of independent samples used was 204.

In the early growth stages of tea chrysanthemums, soil and background interference affected canopy reflectance. This study initially analyzed the correlation between canopy spectra (both with and without soil and background removal) and photosynthetic traits. The results revealed that spectral indices were significantly correlated with photosynthetic traits at a level of 0.05, and showed stronger correlations when the soil background and canopy shadow were removed than when they were not (Fig. 5). Therefore, all subsequent analyses in this study utilized reflectance data with the soil background and canopy shadows removed. Pearson's correlation coefficients between spectral indices and photosynthetic traits (Table S8) demonstrated that most indices were significantly correlated with photosynthetic traits (p ​< ​0.05). Out of 63 evaluated spectral indices, the top ten indices that were correlated with Vcmax were SCCCI, NDRE, CIRE, VIre, CVI, VIg, CI_green_, GNDVI, MCARI2, and NDI (r ​≥ ​0.66), with SCCCI showing the strongest correlation (r ​= ​0.75). The top ten indices for Jmax were CVI, b, GBRI, ExB, IPCA, SCCCI, WI, g, GLI, and ExG (|r| ​≥ ​0.52), with CVI showing the highest correlation (r ​= ​0.66).

### 3.3 Estimation of tea chrysanthemum photosynthetic traits with published spectral indices

**Fig. 6:**
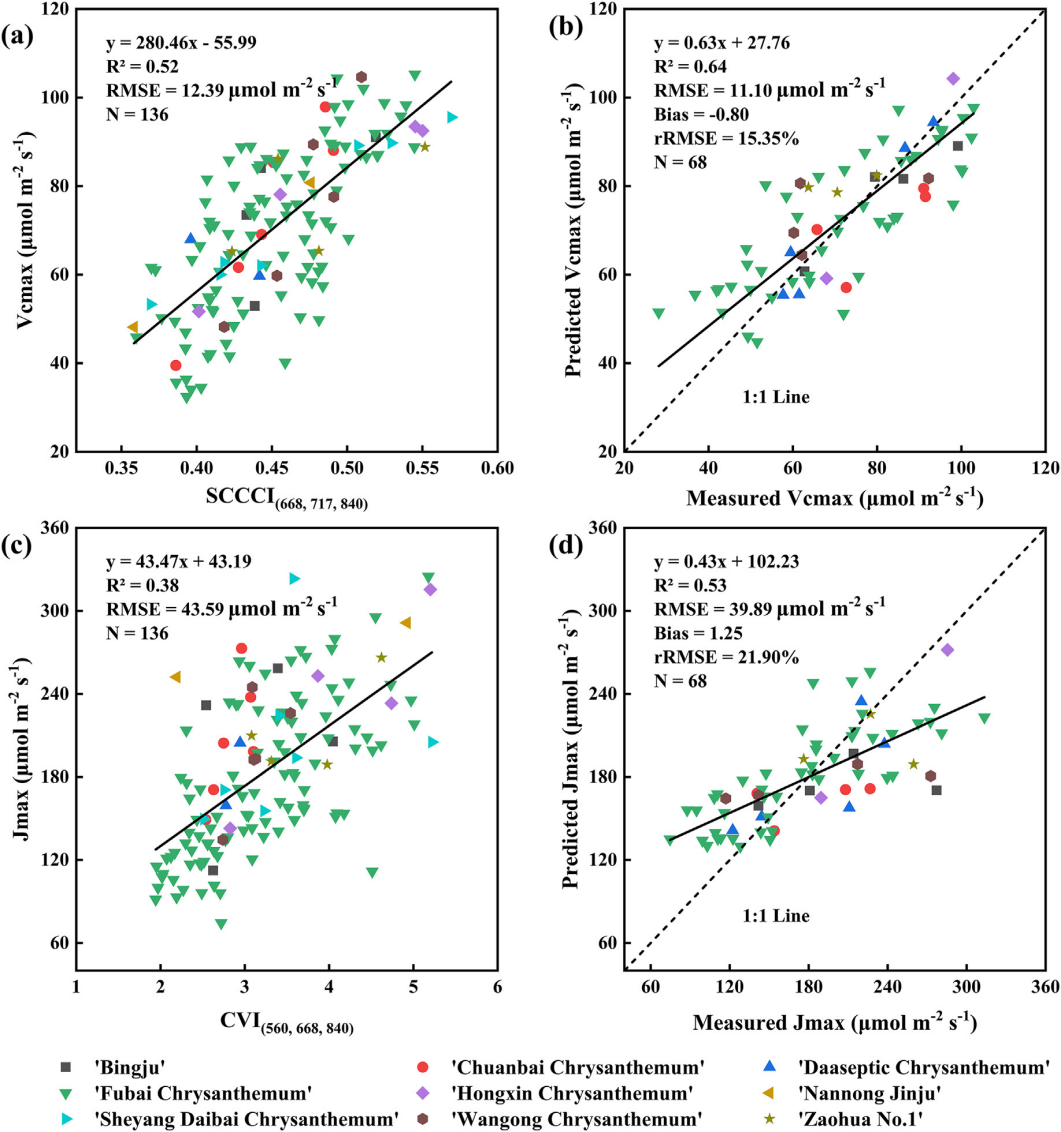
Relationships between photosynthetic traits and optimal spectral indices: (a) SCCCI for Vcmax, (c) CVI for Jmax. Validation results for the estimation of photosynthetic traits using SCCCI (b) and CVI (d).

To evaluate the potential of spectral indices for estimating the photosynthetic traits of tea chrysanthemums, we analyzed the linear relationships between 63 previously published spectral indices related to N, Cab, photosynthetic pigments, and photosynthetic traits. Results showed that the spectral indices most closely related to photosynthetic traits included the green and red-edge bands. The aforementioned best-correlated indices, SCCCI and CVI, were the best-performing indices for estimating Vcmax and Jmax, respectively (Table S9 and S10). For Vcmax, SCCCI achieved an R^2^ of 0.52 and an RMSE of 12.39 ​μmol ​m^−2^ ​s^−1^ for the calibration dataset (Fig. 6a). For the validation dataset, the R^2^, RMSE, Bias, and rRMSE were 0.64, 11.10 ​μmol m-^2^ s^−1^, -0.80 ​μmol ​m^−2^ ​s^−1^, and 15.35 ​%, respectively (Fig. 6b). For Jmax, CVI showed an R^2^ of 0.38 and an RMSE of 43.59 ​μmol ​m^−2^ ​s^−1^ for the calibration dataset (Fig. 6c). For the validation dataset, the R^2^, RMSE, Bias, and rRMSE were 0.53, 39.89 ​μmol ​m^−2^ ​s^−1^, 1.25 ​μmol ​m^−2^ ​s^−1^, and 21.90 ​%, respectively (Fig. 6d). Overall, the spectral index method demonstrated moderate accuracy in estimating the photosynthetic traits of the tea chrysanthemums. The estimation accuracy could be further improved by integrating machine learning algorithms with multiple sensitive spectral indices.

### 3.4 Estimation of tea chrysanthemum photosynthetic traits with machine learning

**Fig. 7:**
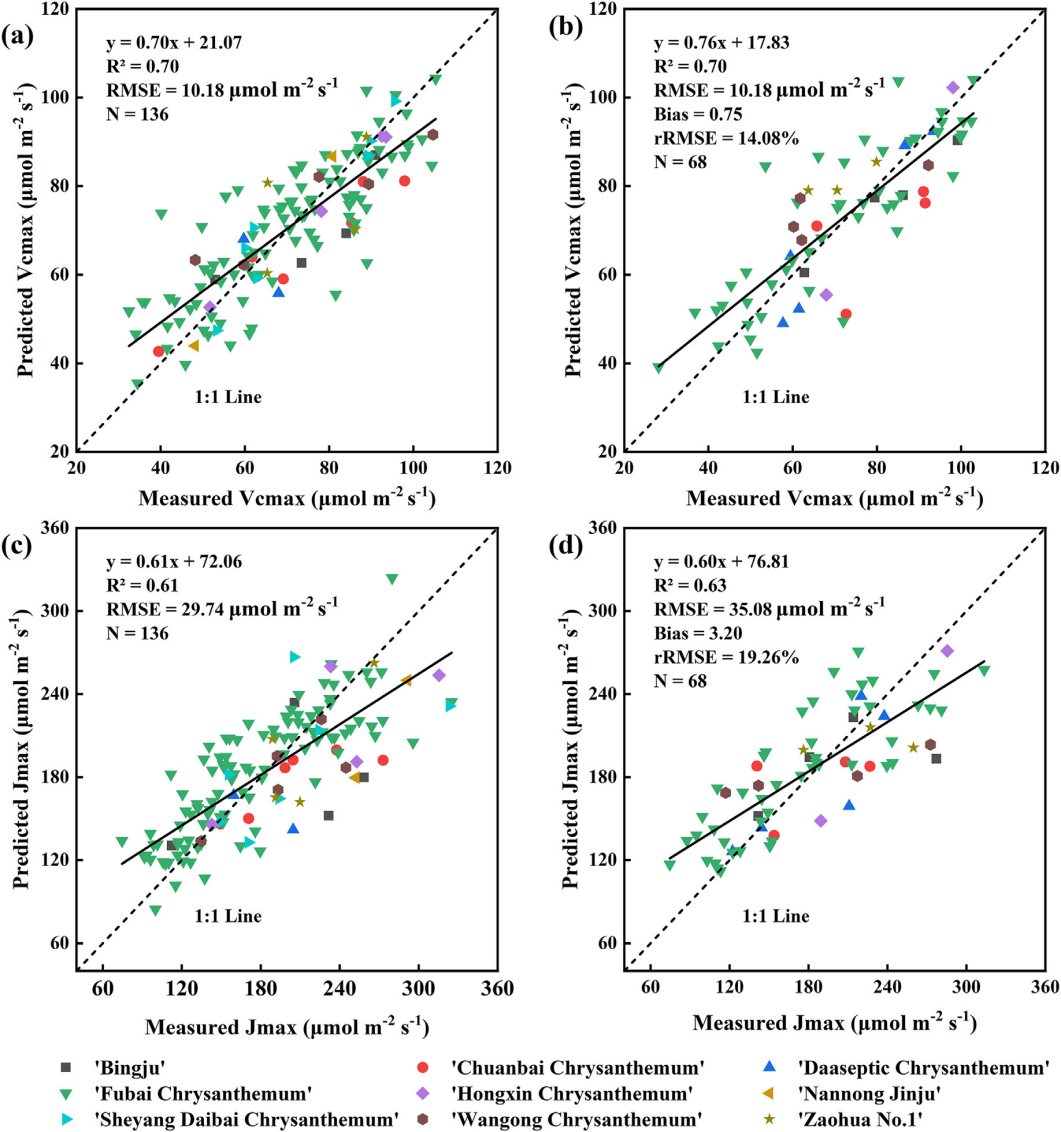
Relationship between the measured and predicted Vcmax and Jmax of tea chrysanthemum using PLSR model: (a) PLSR estimation of Vcmax in the calibration dataset, (b) PLSR estimation of Vcmax in the validation dataset, (c) PLSR estimation of Jmax in the calibration dataset, (d) PLSR estimation of Jmax in the validation dataset. The numbers of components (each component represents a linear combination of the nine or eighteen spectral indices identified by LASSO) determined by 10-fold cross-validation in the PLSR were 7 and 11 for Vcmax and Jmax, respectively.

For this part of analysis, we employed a variable selection algorithm (LASSO) in conjunction with PLSR to predict the photosynthetic traits of tea chrysanthemums. Initially, the LASSO algorithm was used to identify significant spectral indices for estimating these traits, resulting in nine important indices for Vcmax: R, 1/G, 1/NIR, GBRI, IKAW, VARI, RGBVI, SCCCI, and MCARI2. Eighteen indices were identified as significant for estimating Jmax, namely B, R, RE, 1/B, 1/R, 1/RE, 1/NIR, GRRI, IKAW, VARI, MGRVI, VIg, MSAVI2, NDVI, SCCCI, CI2, SIPI, and GNDVI. After selecting these important indices, we used them as independent variables in a machine learning model, with Vcmax and Jmax as dependent variables, to construct PLSR-based models for estimating the photosynthetic traits of tea chrysanthemums. The results indicated that for estimating Vcmax, the R^2^ and RMSE for the modeling dataset were 0.70 and 10.18 ​μmol ​m^−2^ ​s^−1^ (Fig. 7a), while for the validation dataset, R^2^, RMSE, Bias, and rRMSE were 0.70, 10.18 ​μmol ​m^−2^ ​s^−1^, 0.75 ​μmol ​m^−2^ ​s^−1^, and 14.08 ​%, respectively (Fig. 7b). For estimating Jmax, the R^2^ and RMSE for the modeling dataset were 0.61 and 29.74 ​μmol ​m^−2^ ​s^−1^ (Fig. 7c), and for the validation dataset, R^2^, RMSE, Bias, and rRMSE were 0.63, 35.08 ​μmol ​m^−2^ ​s^−1^, 3.20 ​μmol ​m^−2^ ​s^−1^, and 19.26 ​%, respectively (Fig. 7d).

**Fig. 8:**
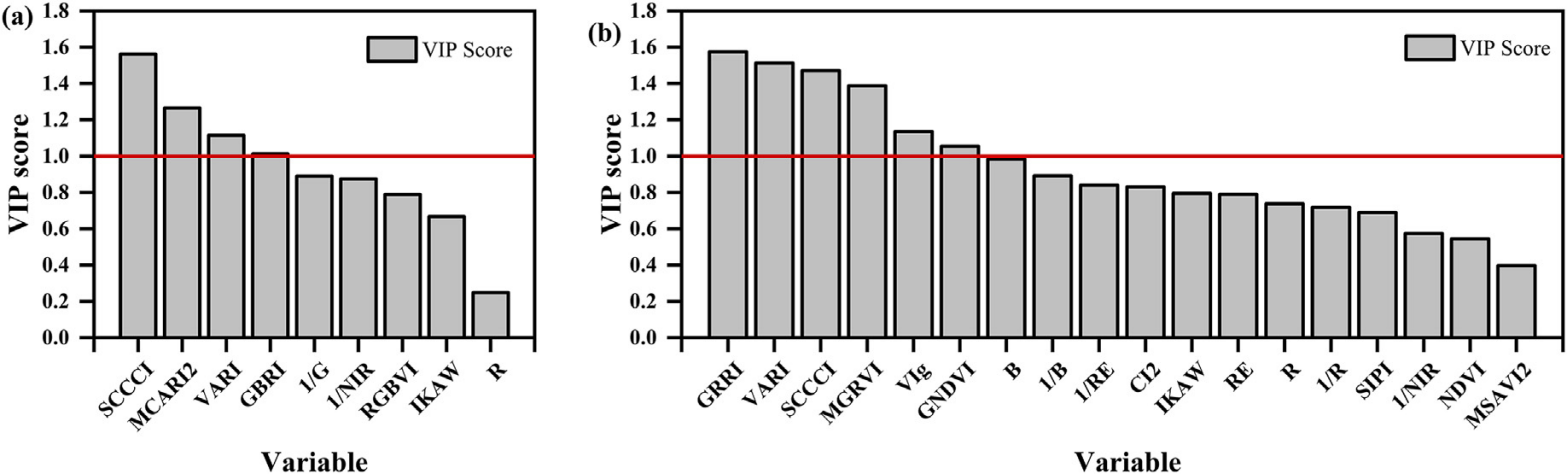
Variable importance in the projection (VIP) of different spectral indices in PLSR models for Vcmax (a) and Jmax (b) inversions.

We analyzed the variable importance in projection (VIP) scores to identify key spectral indices in the PLSR models. The results revealed that in the PLSR model for estimating Vcmax, SCCCI, MCARI2, VARI, and GBRI significantly contributed to the model (Fig. 8a). The most influential indices for estimating Jmax were GRRI, VARI, SCCCI, MGRVI, VIg, and GNDVI (Fig. 8b). Overall, in the PLSR models for estimating the photosynthetic traits of tea chrysanthemums, the spectral indices sensitive to Cab, particularly the red-edge and green bands, had the greatest contribution to the prediction of these traits.

### 3.5 Spatial transferability of the RS approach-photosynthetic traits model in mapping tea chrysanthemum Vcmax and Jmax

**Fig. 9:**
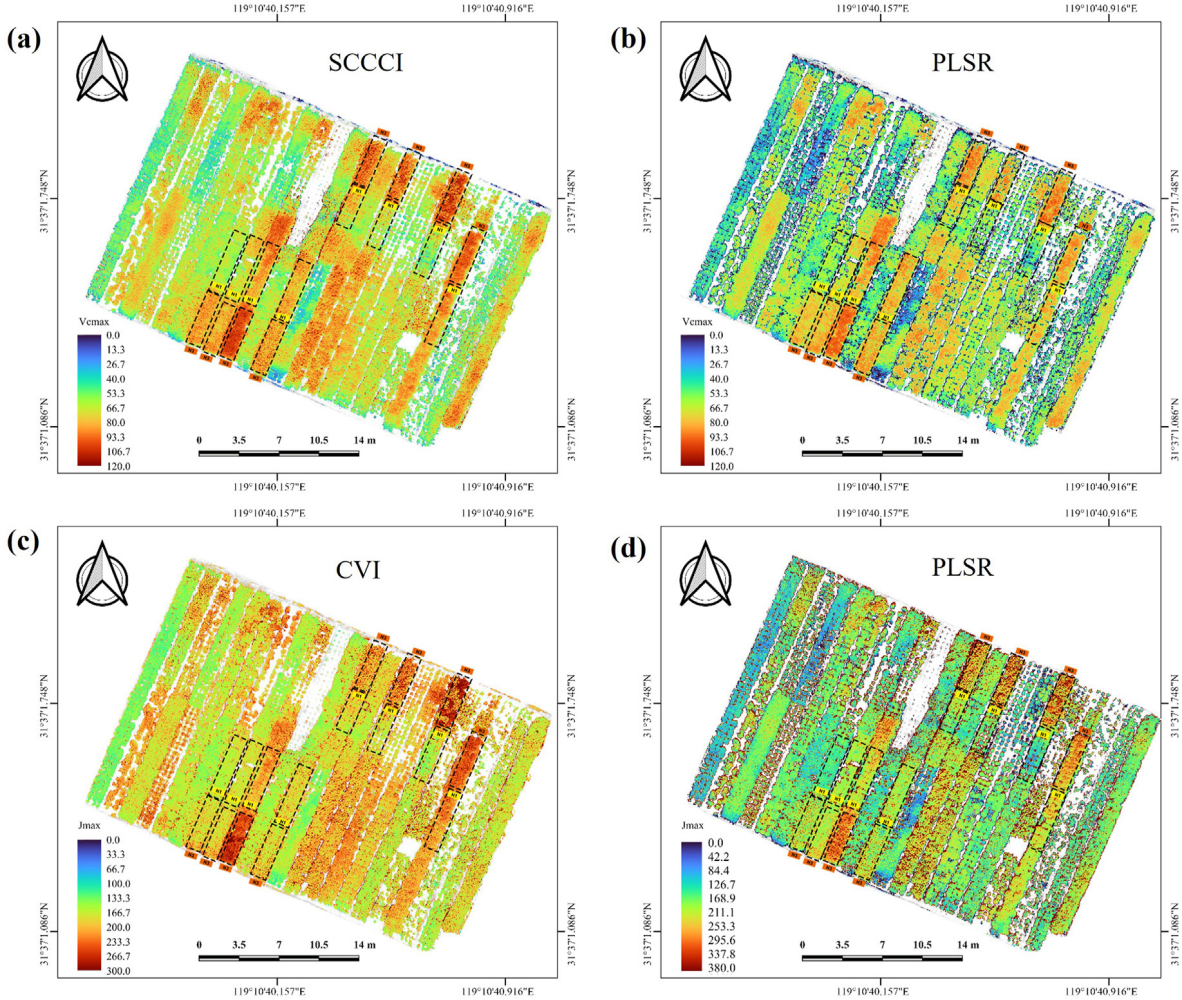
Photosynthetic trait maps produced using RS approaches: (a) Vcmax map generated by the SCCCI spectral index model; (b) Vcmax map generated by the PLSR model; (c) Jmax map generated by the CVI spectral index model; and (d) Jmax map generated by the PLSR model. Images were captured on September 19, 2023. The black dashed rectangles indicate the plots with different nitrogen gradient treatments: 130 ​kg/hm^2^ (N1) and 390 ​kg/hm^2^ (N3).

To evaluate the spatial transferability of the RS-based photosynthetic traits model for mapping tea chrysanthemum Vcmax and Jmax, two RS approaches—spectral index and PLSR—were employed to generate Vcmax and Jmax maps in a multi-variety experiment (Exp. 6). The results demonstrated that both the spectral-index and PLSR based models produced similar spatial patterns. For the spectral index-based model, Vcmax values ranged from 15.46 to 118.59 ​μmol ​m^−2^ ​s^−1^ (Fig. 9a), while the PLSR-based model produced values between 43.06 and 101.53 ​m^−2^ ​s^−1^ (Fig. 9b). In terms of Jmax, the spectral index-based model showed a range of 114.84–297.65 ​μmol ​m^−2^ ​s^−1^ (Fig. 9c), compared to 105.46–254.46 ​μmol ​m^−2^ ​s^−1^ for the PLSR model (Fig. 9d). Overall, compared to PLSR-based photosynthesis models, the photosynthetic traits predicted by spectral index-based models exhibit greater fluctuations in value ranges due to higher RMSE (Fig. 6b–d, Fig. 7b–d and Fig. S10 and Fig. S11).

## 4 Discussion

Our results indicated that spectral indices strongly correlated with photosynthetic traits primarily included the green and red-edge bands (Table S8). This finding aligned with previous studies, which had identified the visible and red-edge regions as the most significant contributors to estimating photosynthetic traits [13,22,23]. The reflectance spectrum between 450 and 680 ​nm is influenced by photosynthetic pigments [50] and carotenoid content [51]. In the ‘red-edge’ region (680–730 ​nm), leaf reflectance increases substantially as chlorophyll absorption diminishes in the near-infrared region [52]. This region has been shown to correlate with photosystem II efficiency (Fv/Fm) [53], Cab [54], and N content [55]. Notably, a significant portion of plant N is allocated to Rubisco, the key enzyme in the Calvin cycle responsible for carbon fixation during photosynthesis. N also directly influences Cab, the essential pigment for capturing light energy. By regulating both Cab and Rubisco activity, N plays a critical role in determining a plant's photosynthetic capacity [56]. Thus, the ability of these bands to accurately estimate crop photosynthetic traits can be attributed to the critical roles of Cab, N, and Rubisco enzymes in photosynthesis, as well as the dominant influence of these pigments on reflectance spectra from 500 to 800 ​nm. The sensitivity of green and red-edge bands, as well as indices combining these bands, to N variation was further evident in the spectral reflectance and representative indices analyzed in this study (Fig. 3, Fig. 4). Additionally, the VIP scores from the PLSR model highlighted that indices most contributing to photosynthetic traits (VIP >1) are those sensitive to Cab or N. These findings corroborated previous studies suggesting that the mechanisms underlying spectral inversion of photosynthetic traits were primarily driven by photosynthetic pigments and N content [14,19]. Moreover, our results demonstrated that the SCCCI index, incorporating red-edge bands, performed best for estimating Vcmax, while the CVI index, which included green light, exceled in estimating Jmax. Both indices were originally developed to estimate crop Cab [57,58]. Compared to other indices containing green or red-edge bands, SCCCI and CVI showed superior performance in estimating photosynthetic traits, likely due to their construction using canopy-scale spectral data, which matches the scale of the data used in this study. Additionally, combined indices, such as the SCCCI, offer enhanced robustness against variations in leaf area index (LAI) and soil background, which can otherwise confound the remote sensing of foliar N and Cab. This concept can be mathematically expressed as the ratio NDRE/NDVI. NDRE is sensitive to foliar pigments, LAI, and soil background, while NDVI primarily captures variability in LAI and soil background. When represented as: $\frac{Foliar\;pigments\times LAI\times Soil\;background}{LAI\times Soil\;background}$, the LAI and soil background components cancel out, isolating a measure directly proportional to foliar pigments. Consequently, SCCCI demonstrated the best performance for estimating photosynthetic traits [59].

Our study utilized spectral indices derived from broad spectral bands (with a width of 10–40 ​nm, Table S5) to estimate photosynthetic traits, which distinguishes our approach from the narrow-band (1–2 ​nm) indices commonly used in previous studies [22,60]. While narrow-band indices are widely employed in controlled environments and laboratory settings, their applicability for operational field monitoring is constrained by sensor limitations and environmental variability. In contrast, broad-band VIs offer several advantages: first, they provide reliable estimates of photosynthetic traits across a wide range of environmental conditions; second, they exhibit greater robustness to environmental fluctuations and sensor errors, making them particularly well-suited for large-scale data collection; and third, they are more practical for operational use in agriculture, enabling efficient and scalable monitoring. These strengths make broad-band VIs more adaptable and valuable for precision agriculture, especially for large-scale, field-based studies.

In this study, the best-performing spectral indices, SCCCI and CVI, estimated photosynthetic traits primarily using information from red-edge and green bands sensitive to Cab. Other indices may incorporate additional information sensitive to photosynthetic pigments, such as the blue band, which detects carotenoids that dissipate excess energy from excited chlorophyll molecules as heat [61] and has also been used to estimate crop photosynthesis [62]. Therefore, we used LASSO for feature selection on the spectral indices. Among the selected indices, several, including the blue light band index, were also identified as important in the VIP analysis. However, the indices selected by LASSO did not completely align with the top correlated indices from correlation analysis (Table S8 and S9). This discrepancy likely arises from collinearity among highly correlated indices and LASSO's distinct feature selection approach. While correlation analysis ranks indices purely based on their individual relationships with photosynthetic traits, LASSO operates in a multivariate context to minimize redundancy and maximize predictive power. Consequently, LASSO may prioritize indices that provide complementary spectral information rather than those with the highest individual correlation coefficients. To mitigate overfitting, LASSO applied coefficient shrinkage, selecting the most relevant features from these correlated indices. Subsequently, we utilized the selected spectral indices, sensitive to photosynthetic traits, in conjunction with machine learning algorithms to estimate photosynthetic traits. Previous studies have shown that among various machine learning algorithms for estimating crop photosynthetic traits, PLSR is the most effective and consistently outperforms other methods [8,10]. As a result, PLSR is widely used in spectral remote sensing studies for estimating photosynthetic traits and is regarded as the optimal machine learning algorithm for this purpose [22,23]. Our results also showed that PLSR outperformed other machine learning models, such as random forest (RF) and support vector machine (SVM) (Fig. S13 and S14). However, most prior research has relied on PLSR models that use full-band reflectance data to estimate photosynthetic traits [13,15]. Such models require high-resolution hyperspectral reflectance data from phenotyping platforms, increasing model complexity and reducing transferability in practical applications. To address these challenges, this study first applied the LASSO algorithm to screen spectral indices and then combined it with PLSR for modeling photosynthetic traits. The results show that the combination of PLSR and LASSO outperformed standalone PLSR models (Table S11). This further demonstrates the effectiveness of LASSO in reducing data dimensionality, lowering model complexity, and improving estimation accuracy. As reported in previous studies, dimensionality reduction helps mitigate multicollinearity, prevent model overfitting, and improve predictive performance to some extent [[44], [45], [46]]. Our findings clearly highlight the potential of incorporating variable selection in machine learning models to predict crop photosynthetic traits effectively.

Our results demonstrated that the established photosynthetic trait estimation model exhibited strong spatial transferability across multi-variety tea chrysanthemums (Fig. 9). This was further supported by the spatial mapping of photosynthetic traits across all experimental trials, where high-nitrogen treatments consistently exhibited higher photosynthetic trait values compared to low-nitrogen treatments (Fig. S5–S9). Beyond nitrogen availability, soil properties and environmental factors such as temperature, humidity, and light intensity from different experiments also influence photosynthetic traits (Fig. S15 and S16) [63]. These factors not only affect plant physiological status but can also alter spectral signals, impacting model prediction accuracy [64]. These factors introduce uncertainty into the prediction of photosynthetic traits, with machine learning algorithms generally exhibiting higher uncertainty than spectral indices (Fig. S12). Future studies could enhance prediction performance by integrating environmental data with spectral information using multi-source data fusion techniques. For example, incorporating temperature and humidity data from remote sensing or ground sensors into machine learning models may improve model generalization. Additionally, multi-temporal data analysis could help capture the dynamic effects of environmental changes on the relationship between spectral features and photosynthetic traits, further strengthening the model's applicability under varying conditions.

Previous studies had demonstrated that hyperspectral full-band reflectance at the leaf scale could effectively estimate crop photosynthetic traits (Vcmax: R^2^ ​≥ ​0.74; Jmax: R^2^ ​≥ ​0.69) [8,16]. Similarly, hyperspectral imagery obtained from ground-based phenotyping platforms had proven effective for estimating crop photosynthetic traits (Vcmax: R^2^ ​≥ ​0.78; Jmax: R^2^ ​≥ ​0.59) [22,23]. Our findings demonstrated that UAV-based phenotyping platforms employing multispectral remote sensing data could also reliably estimate photosynthetic traits (Vcmax: R^2^ ​= ​0.70; Jmax: R^2^ ​= ​0.63). The lower prediction accuracy for Jmax compared to Vcmax can be attributed to several factors. Specifically, Jmax exhibited a higher coefficient of variation (CV ​= ​30.64) than Vcmax (CV ​= ​25.43, Table S6), indicating greater sensitivity to environmental fluctuations during measurement. This finding is consistent with previous studies, which suggest that Jmax, reflecting electron transport chain activity, is more influenced by environmental factors such as light and temperature, whereas Vcmax, associated with Rubisco activity, is less sensitive to these variables [13]. To address this limitation and enhance the prediction accuracy of Jmax, future research could adopt several strategies: increasing the number of measurements to reduce the impact of environmental variability, incorporating additional spectral bands sensitive to Jmax, and integrating environmental data into predictive models. These improvements would not only improve the estimation of Jmax but also contribute to a more comprehensive understanding of crop photosynthetic traits. Additionally, compared to leaf-scale and ground-based phenotyping platforms, the UAV-based platform achieved comparable precision in estimating photosynthetic traits. However, at the canopy scale, factors such as canopy structure and soil background influence reflectance, unlike leaf-scale studies. Therefore, isolating pure leaf pixels is essential for accurate photosynthetic trait estimation at the canopy scale [23]. In our study, the application of the ExG threshold segmentation method to remove soil background and canopy shadow improved estimation accuracy (Fig. 5), corroborating this viewpoint. In this study, the ExG index was manually calculated using the band math tool in ENVI, with its threshold adjusted to remove soil background and canopy shadow interference. Automating this critical step in future work would significantly enhance efficiency and reproducibility.

Regarding time-efficiency and cost, leaf-scale studies generally rely on hand-held spectroradiometers, which require approximately 5 ​s to measure hyperspectral reflectance from a single leaf. Despite their high precision, these devices do not allow to generate spatial maps of photosynthetic traits and are prohibitively expensive, with a market price of around USD 100,000 as of 2024. Ground-based platforms, such as vehicle-mounted [22,23] or tractor-mounted [24] hyperspectral cameras, enabled mapping photosynthetic traits and could acquire a hyperspectral image within approximately 5 ​min. However, these platforms also have high costs (exceeding USD 100,000) and involved complex data processing. Additionally, tractor-mounted systems require traversal of the entire field, which is time-consuming and may cause physical damage to crops, limiting their practicality in densely planted fields. Even multispectral systems like the CropCircle ACS-470 (Holland Scientific Inc., Lincoln, NE, USA), equipped with three bands (green/red, red-edge, and near-infrared), have potential for monitoring photosynthetic traits. However, the system lacks the blue band, which is sensitive to photosynthetic pigments, is priced at USD 15,000, and does not support spatial mapping of photosynthetic traits. In contrast, the UAV-based multispectral phenotyping platform employed in this study offered a significantly lower cost (∼USD 10,000), making it more affordable and accessible compared to hyperspectral sensors. This cost-effectiveness makes the method suitable for large-scale agricultural applications. Additionally, the UAV platform facilitates efficient data acquisition with high spatial and temporal resolution, enabling rapid and repeated monitoring of crop photosynthetic traits across large fields. For instance, it took a single flight of about 5 ​min to capture canopy multispectral imagery of 38 varieties (Exp. 6) while achieving precise spatial mapping with a 2 ​cm ground spatial resolution of crop photosynthetic traits. In summary, the UAV phenotyping platform utilized in this study demonstrated excellent performance in data acquisition efficiency, cost-effectiveness, and photosynthetic trait estimation precision. Consequently, this platform represents an ideal choice for breeders aiming to select crops with high photosynthetic efficiency for phenotypic breeding programs.

In summary, our study makes several key contributions to the field of remote sensing for crop photosynthetic traits. First, we demonstrated the effectiveness of broad-band spectral indices (10–40 ​nm) in estimating photosynthetic traits, offering a more practical and robust alternative to narrow-band indices commonly used in controlled environments. Second, we introduced a novel approach by combining LASSO with PLSR, which not only reduced data dimensionality but also improved model accuracy and transferability. Third, we leveraged a UAV-based multispectral platform to achieve efficient, cost-effective, and precise spatial mapping of photosynthetic traits at a large scale, addressing the limitations of traditional ground-based and leaf-scale methods. These innovations make our approach particularly valuable for precision agriculture and phenotypic breeding programs, where scalability, cost-effectiveness, and robustness are critical. Although this study successfully achieved precise spatial mapping of photosynthetic phenotypes in 38 chrysanthemum varieties, it did not address high-efficiency gene screening. Future research could expand on this work by evaluating a larger population of chrysanthemum varieties—potentially over 200—using the UAV-based multispectral platform developed here. Coupling this approach with genome-wide association studies (GWAS) may enable the identification of key candidate gene loci associated with high photosynthetic efficiency.

## 5 Conclusion

High-throughput, rapid acquisition of photosynthetic phenotypes is essential for identifying high-efficiency photosynthetic genes in crops. This study examined the potential of UAV multispectral remote sensing data for estimating photosynthetic traits in tea chrysanthemums. Results showed that spectral indices from UAV data, particularly those incorporating green or red-edge bands, showed the strongest correlations with photosynthetic traits. Specifically, the SCCCI and CVI were the most effective indices for estimating Vcmax and Jmax, respectively. Furthermore, the integration of machine learning with spectral feature selection algorithms significantly enhanced estimation accuracy and enabled more precise spatial mapping of photosynthetic traits. Our findings will offer a novel technical approach for high-throughput acquisition of crop photosynthetic phenotype information.

## CRediT authorship contribution statement

**Jingshan Lu:** Conceptualization, Data curation, Formal analysis, Investigation, Methodology, Software, Validation, Visualization, Writing – original draft, Writing – review & editing. **Qimo Qi:** Conceptualization, Data curation, Formal analysis, Investigation, Methodology, Software, Validation, Visualization, Writing – original draft, Writing – review & editing. **Gangjun Zheng:** Conceptualization, Data curation, Formal analysis, Investigation, Methodology, Software, Validation, Visualization, Writing – original draft, Writing – review & editing. **Jan U.H. Eitel:** Data curation, Writing – original draft, Writing – review & editing. **Qiuyan Zhang:** Data curation, Writing – original draft, Writing – review & editing. **Jiuyuan Zhang:** Data curation, Writing – original draft, Writing – review & editing. **Sumei Chen:** Supervision, Writing – original draft. **Fei Zhang:** Supervision, Writing – original draft. **Weimin Fang:** Supervision, Writing – original draft. **Zhiyong Guan:** Conceptualization, Resources, Writing – original draft, Writing – review & editing, Supervision, Project administration, Funding acquisition. **Fadi Chen:** Conceptualization, Resources, Writing – original draft, Writing – review & editing, Supervision, Project administration, Funding acquisition.

## Data availability

The calibration and validation datasets of UAV-based spectral indices and photosynthesis supporting our results are available in the GitHub repositories at https://github.com/ljs19930709/UAV-and-Photosynthesis-dataset-.git. Other data are openly available from the corresponding author upon reasonable request.

## Funding

This work was supported by the 10.13039/501100001809National Natural Science Foundation of China (32302593), 10.13039/501100002949Natural Science Fund of Jiangsu Province (BK20230996), the Jiangsu Funding Program for Excellent Postdoctoral Talent (2022ZB339), the Fellowship of 10.13039/501100002858China Postdoctoral Science Foundation (2022M721638), 10.13039/501100002949The“JBGS”Project of Seed Industry Revitalization in Jiangsu Province (JBGS (2021) 020), the Program for Key Research and Development, Jiangsu, China (Modern Agriculture) (BE2023367), the earmarked fund for 10.13039/501100020051Jiangsu Agricultural Industry Technology System. We are grateful to the editor and three anonymous reviewers for their suggestions and comments, which significantly improved the quality of this paper.

## Declaration of competing interest

The authors declare that they have no known competing financial interests or personal relationships that could have appeared to influence the work reported in this paper.
