# Supplementary material for: High-Throughput Field Phenotyping Using Unmanned Aerial Vehicles (UAVs) for Rapid Estimation of Photosynthetic Traits
Source: Plant Phenomics. 2025 Apr 26;7(2):100045. doi: 10.1016/j.plaphe.2025.100045 (PMC12710006; doi:10.1016/j.plaphe.2025.100045)
Supplement: Multimedia component 1 [file mmc1.docx]

**Supplementary Materials**

**Table S1** Overview of tea chrysanthemum experiments.

| **Experiments**  **(Exp.)** | **Year** | **Site** | **Chrysanthemum Varieties** | **N** **gradient treatments** | **Planting date** |
| --- | --- | --- | --- | --- | --- |
| Exp. 1 | 2022 | Qixia | ‘Fubai Chrysanthemum’ | N0, N1, N2, N3, N4 | 6/15 |
| Exp. 2 | 2022 | Qixia | ‘Nannong Jinju’ | N0, N1, N2, N3, N4 | 6/15 |
| Exp. 3 | 2022 | Lishui | ‘Fubai Chrysanthemum’ | N0, N1, N2, N3, N4 | 6/15 |
| Exp. 4 | 2023 | Qixia | ‘Fubai Chrysanthemum’ | N0, N1, N2, N3, N4 | 5/25 |
| Exp. 5 | 2023 | Lishui | ‘Fubai Chrysanthemum’ | N0, N1, N2, N3, N4 | 5/25 |
| Exp. 6 | 2023 | Lishui | ‘Hongxin Chrysanthemum’,  ‘Daaseptic Chrysanthemum’, ‘Sheyang Daibai Chrysanthemum’, ‘Wangong Chrysanthemum’, ‘Zaohua No.1’, ‘Chuanbai Chrysanthemum’, ‘Bingju’ | N1, N3 | 5/25 |

**Note:** N0 =0 kg/hm², N1 =130 kg/hm², N2 =260 kg/hm², N3 = 390 kg/hm², N4 =520 kg/hm², nitrogen fertilizer = urea (N≥46%). The varieties listed in Exp. 6 column of the table are those tested for the A-Ci response curves. The 38 varieties are shown in Table S2 and Fig. S1.

**Table S2** Multiple varieties of tea chrysanthemum in the experiment 6.

| Number | Cultivar | Number | Cultivar | Number | Cultivar |
| --- | --- | --- | --- | --- | --- |
| C1 | ‘Nannong Jinju’ | **C14** | **‘Chuanbai Chrysanthemum’** | C27 | ‘Huangzao Xiangju’ |
| C2 | ‘Qiyue Bai’ | C15 | ‘Tao Chrysanthemum’ | C28 | ‘Huangwan Xiangju’ |
| C3 | ‘Hangbai Chrysanthemum' | **C16** | **‘Zaohua No.1'** | C29 | ‘Baiwan Xiangju’ |
| C4 | ‘Xiaoxiangju Bai' | C17 | ‘Jinju No.2’ | **C30** | **‘Hongxin Chrysanthemum’** |
| C5 | ‘Xiuning Dabaihua' | C18 | ‘Jinju No.3’ | C31 | ‘Yanming Le’ |
| C6 | ‘Chu Chrysanthemum' | **C19** | **‘Wangong Chrysanthemum '** | C32 | ‘Nannong Lixiang’ |
| C7 | ‘Xiaoxiangju Huang’ | **C20** | **‘Sheyang Dabaiju'** | C33 | ‘Hebei Xiangju’ |
| C8 | ‘Dayanghua’ | C21 | ‘Xiaohuangju’ | C34 | ‘Huangjin Chrysanthemum’ |
| C9 | ‘Xiaoyanghua’ | C22 | ‘Jining Jinju Huang’ | C35 | ‘Suju No.13’ |
| C10 | ‘Eryanghua’ | C23 | ‘Jining Jinju Bai’ | C36 | ‘Jinsi Huangju’ |
| C11 | ‘Qiyue Huang’ | C24 | **‘Da Aseptic Chrysanthemum’** | C37 | ‘Gong Chrysanthemum’ |
| **C12** | **‘Bingju’** | C25 | ‘Xiao Aseptic Chrysanthemum’ | C38 | ‘Fubai Chrysanthemum’ |
| C13 | ‘Xiuning Xiangju’ | C26 | ‘Daguo Chrysanthemum’ |  |  |

**Note:** Bold indicates the varieties used to measure the A-Ci response curves.

**Table S3** Dates of field data collection.

| **Experiments (Exp.)** | **UAV flight and A-Ci response curve measurement dates** | **Crop growth stages associated**  **with the dates** | **Number of A-Ci curve samples (N)** |
| --- | --- | --- | --- |
| Exp. 1 | 9/17, 9/27, 10/12, 10/25 | Middle branching, late branching, initial budding, middle budding | 16 |
| Exp. 2 | 9/27 | Late branching | 2 |
| Exp. 3 | 9/18, 10/4, 10/11, 10/21 | Middle branching, late branching, initial budding, middle budding | 37 |
| Exp. 4 | 8/19, 9/28, 10.11 | Initial branching, late branching, initial budding | 43 |
| Exp. 5 | 8/29, 8/31, 9/18, 10/9 | Initial branching, initial branching, middle branching, initial budding | 52 |
| Exp. 6 | 9/1, 9/19, 9/25, 9/27, 10/10 | Initial branching, middle branching, late branching, late branching, initial budding | 54 |

**Note:** Measurement of A-Ci response curve in EXP. 6 cover 7 varieties including ‘Hongxin Chrysanthemum’, ‘Daaseptic Chrysanthemum’, ‘Sheyang Daibai Chrysanthemum’, ‘Wangong Chrysanthemum’, ‘Zaohua No.1’, ‘Chuanbai Chrysanthemum’, ‘Bingju’.

**Table S4** Specific parameters of DJI Inspire 2 UAV.

| **Detailed parameters** | **Value** |
| --- | --- |
| Weight (including propellers and two batteries) | 3440 g |
| Front obstacle avoidance | 30 m |
| Maximum horizontal flight speed | 94 km/h |
| Maximum allowable wind speed | 10 m/s |
| Maximum allowable wind speed | P mode/A mode: 4 m/s; S mode: 6 m/s |
| Maximum descent speed | Vertical: 4 m/s; Oblique descent: 4 - 9 m/s |
| Operating temperature range | -20 - 40 ℃ |

**Table S5** The specific parameters of the five bands of the RedEdge-MX camera.

| **Spectral wavelength name** | **Center reflected wavelength** | **Spectral width** |
| --- | --- | --- |
| Band 1: Blue band (B) | 475 nm | 20 nm |
| Band 2: Green band (G) | 560 nm | 20 nm |
| Band 3: Red band (R) | 668 nm | 10 nm |
| Band 4: Red-edge band (RE) | 717 nm | 10 nm |
| Band 5: Near-infrared band (NIR) | 840 nm | 40 nm |

**Table S6** Descriptive statistics of photosynthetic traits in tea chrysanthemum from different datasets.

| Dataset | Number  of sample | Vcmax (μmol m^-2^ s^-1^) | | | | |  | Jmax (μmol m^-2^ s^-1^) | | | | | |
| --- | --- | --- | --- | --- | --- | --- | --- | --- | --- | --- | --- | --- | --- |
|  |  | Min | Max | Mean | STD | CV（%） |  | Min | Max | Mean | STD | CV（%） |  |
| Exp. 1 | 16 | 71.19 | 100.19 | 86.15 | 10.11 | 11.74% |  | 147.64 | 313.51 | 225.25 | 44.29 | 19.66% |  |
| Exp. 2 | 2 | 48.14 | 80.80 | 64.47 | 23.09 | 35.82% |  | 252.18 | 291.42 | 271.80 | 27.75 | 10.21% |  |
| Exp. 3 | 37 | 56.55 | 104.37 | 83.48 | 10.72 | 12.85% |  | 96.23 | 324.98 | 202.44 | 49.88 | 24.64% |  |
| Exp. 4 | 43 | 28.15 | 105.27 | 63.12 | 21.22 | 33.62% |  | 74.63 | 281.05 | 149.94 | 50.52 | 33.69% |  |
| Exp. 5 | 52 | 32.45 | 97.99 | 62.24 | 15.21 | 24.44% |  | 74.54 | 272.77 | 160.71 | 50.23 | 31.26% |  |
| Exp. 6 | 54 | 39.49 | 104.65 | 74.97 | 15.84 | 21.12% |  | 112.43 | 323.36 | 203.43 | 49.97 | 24.57% |  |
| N0 | 47 | 28.15 | 88.98 | 53.41 | 15.08 | 28.24% |  | 74.54 | 252.18 | 132.14 | 42.69 | 32.31% |  |
| N1 | 13 | 69.23 | 98.82 | 81.59 | 8.03 | 9.84% |  | 147.55 | 291.42 | 202.94 | 48.12 | 23.71% |  |
| N2 | 41 | 40.16 | 92.62 | 66.81 | 12.83 | 19.20% |  | 110.74 | 254.80 | 166.47 | 43.21 | 25.96% |  |
| N3 | 12 | 79.09 | 104.37 | 91.05 | 7.03 | 7.73% |  | 183.41 | 275.47 | 231.49 | 29.08 | 12.56% |  |
| N4 | 37 | 58.33 | 105.27 | 84.97 | 12.89 | 15.17% |  | 140.77 | 324.98 | 215.96 | 48.54 | 22.48% |  |
| Calibration | 136 | 32.45 | 105.27 | 71.16 | 18.03 | 25.33% |  | 74.54 | 324.98 | 184.13 | 55.71 | 30.25% |  |
| Validation | 68 | 28.15 | 102.90 | 72.31 | 18.64 | 25.77% |  | 74.63 | 313.51 | 182.16 | 57.63 | 31.64% |  |
| All | 204 | 28.15 | 105.27 | 71.54 | 18.19 | 25.43% |  | 74.54 | 324.98 | 183.47 | 56.22 | 30.64% |  |

**Table S7** Published spectral indices used in this study.

| **Spectral indices** | **Sensitive traits** | **References** |
| --- | --- | --- |
| B = R_B_ | Carotenoid | / |
| G = R_G_ | Cab | / |
| R = R_R_ | Cab | / |
| RE = R_RE_ | Cab and N | / |
| NIR = R_NIR_ | / | / |
| 1/B = 1/B | / | / |
| 1/G = 1/G | / | / |
| 1/R = 1/R | / | / |
| 1/RE = 1/RE | / | / |
| 1/NIR = 1/NIR | / | / |
| lnB = lnB | / | / |
| lnG = lnG | / | / |
| lnR = lnR | / | / |
| lnRE = lnRE | / | / |
| lnNIR = lnNIR | / | / |
| r = R/(R+G+B) | Photosynthesis | [1] |
| g = G/(R+G+B) | Photosynthesis | [1] |
| b = B/(R+G+B) | Photosynthesis | [2] |
| GRRI = G/R | Cab | [2] |
| GBRI = G/B | Cab | [2] |
| RBRI = R/B | Cab | [2] |
| INT = (R+G+B)/3 | Cab | [3] |
| GRVI = (G–R)/(G+R) | GPP and N | [4] |
| NDI = (r–g)/(r+g+0.01) | Cab | [5] |
| WI = (G–B)/(G+R) | Cab | [6] |
| IKAW = (R–B)/(R+B) | Cab and N | [7] |
| GLI = (2×G–R–B)/(2×G+R+B) | Cab | [8] |
| GLI2 = (2×G–R+B)/(2×G+R+B) | Cab | [8] |
| VARI = (G–R)/(G+R–B) | Cab and N | [9] |
| ExR = 1.4×r–g | Cab | [10] |
| ExG = 2×g–r–b | Cab | [10] |
| ExB = 1.4×b–g | Cab | [10] |
| ExGR = ExG–ExR | Cab | [10] |
| IPCA = 0.994×R–B+0.961×G–B+0.914×G–R | Cab | [11] |
| RGBVI = (G^2^–B×R)/(G^2^+B×R) | Cab | [12] |
| MGRVI = (G^2^–R^2^)/(G^2^+R^2^) | Cab | [13] |
| VIg = NIR/G | Cab | [14] |
| VIr = NIR/R | Cab | [15] |
| VIre = NIR/RE | Cab and N | [15] |
| ln_re_ = 100×(ln_NIR_–ln_R_) | Cab | [16] |
| MSAVI = (1+L)($\frac{NIR-R}{NIR+R+L}$)(L = 0.1) | Cab | [17] |
| MSAVI2 = NIR+0.5–$\sqrt{{(2\times NIR+1)}^{2}-8\times(NIR-R)/2}$ | Cab | [17] |
| OSAVI = (1+0.16)×(NIR–R)/(NIR+R+0.16) | Cab and N | [18] |
| MSR = (NIR–R–1)/($\sqrt{NIR+R}$+1) | Cab | [19] |
| MTVI2 = $\frac{1.5\times[1.2\times\left( NIR-G \right)-2.5\times(R-G)}{\sqrt{{(2\times NIR+1)}^{2}-\left( 6\times NIR-5\times\sqrt{R} \right)-0.5}}$ | Cab and N | [20] |
| SAVI = $\frac{(NIR-R)}{(NIR+R+0.5)}$×(1+0.5) | Cab | [21] |
| NDRE = (NIR–RE)/(NIR+RE) | Cab and N | [22] |
| NDVI = (NIR–R)/(NIR+R) | Cab | [23] |
| SCCCI = NDRE/NDVI | Cab | [24] |
| MCARI = (RE–R–0.2×(RE-G)) ×(RE/R) | Cab and N | [25] |
| MCARI2 = 1.5×$\frac{(2.5\times\left( NIR-RE \right)-1.3\times\left( NIR-G \right))}{(2\times\left( NIR+1 \right)^{2}-\left( 6\times NIR-5\times R^{2} \right)-0.5)}$ | Cab and N | [20] |
| TCARI = 3×((RE-R)-0.2×(RE-G) ×(RE/R)) | Cab | [26] |
| CI2 = RE/G–1 | Cab and N | [27] |
| SIPI = (NIR-G)/(NIR+R) | Cab, N, and carotenoid | [28] |
| CI_Green_ = NIR/G–1 | Cab and N | [29] |
| CI_RE_ = NIR/RE–1 | Cab and N | [29] |
| DVI = NIR–R | Cab | [30] |
| RVI = NIR/R | Cab | [31] |
| GNDVI = (NIR–G)/(NIR+G) | Cab and N | [32] |
| CVI = NIR×R/G^2^ | Cab | [33] |
| LCI = (NIR–RE)/(NIR+R) | Cab and N | [34] |
| EVI = 2.5×(NIR–R)/(NIR+6×R-7.5×B+1) | Biomass | [35] |
| TVI = 0.5×(120×(NIR–G)–200×(R–G)) | Cab and N | [36] |

**Note:** R_B_, R_G_, R_R_, R_RE_, R_NIR_, and GPP are blue, green, red, red-edge, near-infrared band reflectance values, and gross primary productivity, respectively.

**Table S8** Correlation coefficient (r) (linear model) between photosynthetic traits and published spectral indices.

| Spectral indices | Vcmax | Jmax | Spectral indices | Vcmax | Jmax | Spectral indices | Vcmax | Jmax |
| --- | --- | --- | --- | --- | --- | --- | --- | --- |
| B | 0.12^ns^ | 0.28^***^ | INT | -0.14^*^ | 0.00^ns^ | OSAVI | 0.44^***^ | 0.18^*^ |
| G | -0.37^***^ | -0.30^***^ | GRVI | -0.29^***^ | -0.45^***^ | MTVI2 | 0.35^***^ | 0.09^ns^ |
| R | 0.02^ns^ | 0.19^**^ | NDI | 0.66^***^ | 0.43^***^ | MSR | 0.48^***^ | 0.27^***^ |
| RE | -0.16^*^ | -0.17^*^ | WI | -0.39^***^ | -0.54^***^ | SAVI | 0.47^***^ | 0.23^***^ |
| NIR | 0.47^***^ | 0.27^***^ | IKAW | -0.29^***^ | -0.22^**^ | NDRE | 0.70^***^ | 0.48^***^ |
| 1/B | -0.11^ns^ | -0.25^***^ | GLI | -0.36^***^ | -0.52^***^ | NDVI | 0.23^***^ | -0.02^ns^ |
| 1/G | 0.39^***^ | 0.33^***^ | GLI2 | -0.26^***^ | -0.42^***^ | SCCCI | 0.75^***^ | 0.56^***^ |
| 1/R | 0.03^ns^ | -0.14^ns^ | VARI | -0.48^***^ | -0.49^***^ | MCARI | -0.16^*^ | -0.35^***^ |
| 1/RE | 0.15^*^ | 0.18^*^ | ExR | 0.31^***^ | 0.47^***^ | MCARI2 | 0.66^***^ | 0.43^***^ |
| 1/NIR | -0.50^***^ | -0.30^***^ | ExG | -0.37^***^ | -0.52^***^ | TCARI | 0.00^ns^ | 0.22^**^ |
| lnB | 0.11^ns^ | 0.27^***^ | ExB | 0.42^***^ | 0.56^***^ | CI2 | 0.44^***^ | 0.31^***^ |
| lnG | -0.38^***^ | -0.32^***^ | ExGR | -0.35^***^ | -0.50^***^ | SIPI | -0.25^***^ | -0.08^ns^ |
| lnR | 0.00^ns^ | 0.17^*^ | IPCA | -0.53^***^ | -0.56^***^ | CI_Green_ | 0.68^***^ | 0.48^***^ |
| lnRE | -0.15^*^ | -0.18^*^ | RGBVI | -0.34^***^ | -0.51^***^ | CI_RE_ | 0.70^***^ | 0.48^***^ |
| lnNIR | 0.49^***^ | 0.29^***^ | MGRVI | -0.27^***^ | -0.43^***^ | DVI | 0.47^***^ | 0.25^***^ |
| r | 0.24^***^ | 0.40^***^ | VIg | 0.68^***^ | 0.48^***^ | RVI | 0.31^***^ | 0.06^ns^ |
| g | -0.37^***^ | -0.52^***^ | VIr | 0.31^***^ | 0.06^ns^ | GNDVI | 0.67^***^ | 0.46^***^ |
| b | 0.47^***^ | 0.59^***^ | VIre | 0.70^***^ | 0.48^***^ | CVI | 0.69^***^ | 0.66^***^ |
| GRRI | -0.31^***^ | -0.47^***^ | lnre | 0.28^***^ | 0.02^ns^ | LCI | 0.66^***^ | 0.43^***^ |
| GBRI | -0.46^***^ | -0.58^***^ | MSAVI | 0.41^***^ | 0.14^*^ | EVI | 0.49^***^ | 0.26^***^ |
| RBRI | -0.29^***^ | -0.22^**^ | MSAVI2 | 0.48^***^ | 0.23^**^ | TVI | 0.43^***^ | 0.21^**^ |

**Note:** Significance level: ns not significant, *p < 0.05, **p < 0.01, ***p < 0.001. The number of sample used in this analysis was 204. Grey box represents that the spectral index has top ten performance for photosynthetic traits estimates.

**Table S9** Relationships (linear model) between Vcmax and published spectral indices.

| Spectral  indices | Coefficient | Intercept | Calibration | | Validation | | | |
| --- | --- | --- | --- | --- | --- | --- | --- | --- |
|  |  |  | R² | RMSE  (μmol m^-2^ s^-1^) | R² | RMSE  (μmol m^-2^ s^-1^) | Bias  (μmol m^-2^ s^-1^) | rRMSE |
| SCCCI | 280.446 | -55.99 | 0.52 | 12.39 | 0.64 | 11.10 | -0.80 | 15.35% |
| CVI | 15.26 | 21.69 | 0.45 | 13.31 | 0.54 | 12.87 | 2.28 | 17.80% |
| VIre | 39.75 | -23.84 | 0.42 | 13.71 | 0.63 | 11.53 | -1.36 | 15.94% |
| NDRE | 235.74 | -24.49 | 0.42 | 13.68 | 0.64 | 11.47 | -1.27 | 15.86% |
| CIRE | 39.75 | 15.91 | 0.42 | 13.71 | 0.63 | 11.53 | -1.36 | 15.94% |
| VIg | 7.81 | 10.71 | 0.38 | 14.19 | 0.64 | 11.71 | -0.34 | 16.19% |
| CIGreen | 7.81 | 18.52 | 0.38 | 14.19 | 0.64 | 11.71 | -0.34 | 16.19% |
| GNDVI | 301.11 | -159.35 | 0.37 | 14.27 | 0.64 | 11.68 | -0.28 | 16.15% |
| NDI | 201.41 | -38.70 | 0.36 | 14.41 | 0.62 | 11.99 | -1.26 | 16.58% |
| LCI | 201.41 | -38.70 | 0.36 | 14.41 | 0.62 | 11.99 | -1.26 | 16.58% |
| MCARI2 | 106.12 | 45.37 | 0.34 | 14.54 | 0.62 | 11.85 | -0.50 | 16.39% |
| IPCA | -672.33 | 126.90 | 0.30 | 14.99 | 0.24 | 16.14 | 0.09 | 22.32% |
| VARI | -9908.10 | 100.09 | 0.26 | 15.47 | 0.19 | 16.68 | -0.27 | 23.07% |
| b | 447.53 | -10.53 | 0.23 | 15.78 | 0.24 | 16.44 | 2.44 | 22.73% |
| GBRI | -14.60 | 118.23 | 0.22 | 15.91 | 0.22 | 16.65 | 2.49 | 23.02% |
| ExB | 111.69 | 107.15 | 0.18 | 16.25 | 0.19 | 17.01 | 2.77 | 23.52% |
| 1/G | 3.32 | 20.71 | 0.16 | 16.42 | 0.13 | 17.29 | -0.45 | 23.91% |
| lnG | -49.47 | -62.89 | 0.16 | 16.49 | 0.13 | 17.32 | -0.63 | 23.95% |
| WI | -109.70 | 124.05 | 0.16 | 16.49 | 0.17 | 17.28 | 2.80 | 23.90% |
| G | -704.26 | 118.52 | 0.15 | 16.60 | 0.13 | 17.36 | -0.74 | 24.01% |
| 1/NIR | -25.03 | 121.00 | 0.15 | 16.55 | 0.49 | 14.29 | 1.87 | 19.76% |
| lnNIR | 47.88 | 103.67 | 0.14 | 16.67 | 0.48 | 14.46 | 1.73 | 20.00% |
| g | -154.24 | 160.28 | 0.14 | 16.65 | 0.15 | 17.46 | 2.77 | 24.14% |
| GLI | -92.68 | 114.06 | 0.14 | 16.69 | 0.15 | 17.50 | 2.79 | 24.20% |
| ExG | -51.41 | 108.87 | 0.14 | 16.65 | 0.15 | 17.46 | 2.77 | 24.14% |
| CI2 | 25.21 | 15.10 | 0.14 | 16.61 | 0.35 | 15.77 | 1.32 | 21.81% |
| ExGR | -30.07 | 100.50 | 0.13 | 16.78 | 0.13 | 17.61 | 2.73 | 24.35% |
| MSR | 129.83 | 110.00 | 0.13 | 16.76 | 0.48 | 14.59 | 1.49 | 20.18% |
| EVI | 79.64 | 8.13 | 0.13 | 16.72 | 0.52 | 14.37 | 1.13 | 19.87% |
| NIR | 88.17 | 26.00 | 0.12 | 16.80 | 0.47 | 14.73 | 1.59 | 20.37% |
| RGBVI | -79.06 | 131.20 | 0.12 | 16.81 | 0.15 | 17.58 | 2.83 | 24.31% |
| MSAVI2 | 87.86 | 3.95 | 0.12 | 16.80 | 0.51 | 14.64 | 0.82 | 20.24% |
| SAVI | 108.82 | -4.33 | 0.12 | 16.85 | 0.50 | 14.68 | 1.02 | 20.30% |
| DVI | 85.98 | 29.57 | 0.12 | 16.84 | 0.47 | 14.75 | 1.34 | 20.40% |
| GRRI | -12.99 | 103.14 | 0.10 | 17.00 | 0.09 | 17.87 | 2.40 | 24.71% |
| ExR | 70.59 | 88.26 | 0.10 | 16.99 | 0.10 | 17.84 | 2.64 | 24.67% |
| GRVI | -67.78 | 99.10 | 0.09 | 17.12 | 0.09 | 17.97 | 2.59 | 24.85% |
| OSAVI | 131.09 | -33.39 | 0.09 | 17.09 | 0.50 | 15.26 | 0.56 | 21.10% |
| TVI | 1.22 | 34.00 | 0.09 | 17.12 | 0.45 | 15.34 | 1.30 | 21.21% |
| GLI2 | -110.48 | 147.95 | 0.08 | 17.26 | 0.07 | 18.12 | 2.51 | 25.06% |
| MGRVI | -48.39 | 104.82 | 0.08 | 17.22 | 0.09 | 18.06 | 2.62 | 24.97% |
| MSAVI | 132.56 | -38.56 | 0.08 | 17.25 | 0.49 | 15.75 | 0.39 | 21.78% |
| r | 162.94 | 32.11 | 0.07 | 17.36 | 0.05 | 18.23 | 2.39 | 25.21% |
| RE | -204.87 | 115.18 | 0.06 | 17.39 | 0.00 | 19.14 | -0.50 | 26.47% |
| 1/RE | 9.18 | 27.98 | 0.06 | 17.41 | 0.01 | 19.21 | -0.43 | 26.56% |
| lnRE | -43.72 | 3.71 | 0.06 | 17.39 | 0.00 | 19.17 | -0.47 | 26.51% |
| RBRI | -37.90 | 121.05 | 0.06 | 17.44 | 0.18 | 17.39 | 0.40 | 24.05% |
| IKAW | -105.14 | 85.30 | 0.06 | 17.40 | 0.17 | 17.36 | 0.45 | 24.01% |
| MCARI | -13.30 | 87.84 | 0.06 | 17.45 | 0.00 | 18.90 | 1.21 | 26.14% |
| VIr | 0.80 | 55.94 | 0.05 | 17.52 | 0.29 | 17.00 | 0.30 | 23.51% |
| MTVI2 | 48.76 | 31.90 | 0.05 | 17.52 | 0.42 | 16.47 | 0.73 | 22.78% |
| RVI | 0.80 | 55.94 | 0.05 | 17.52 | 0.29 | 17.00 | 0.30 | 23.51% |
| lnre | 0.12 | 36.89 | 0.03 | 17.66 | 0.31 | 17.32 | 0.31 | 23.95% |
| SIPI | -350.85 | 427.14 | 0.03 | 17.73 | 0.37 | 17.61 | 0.36 | 24.35% |
| INT | -365.97 | 85.45 | 0.02 | 17.79 | 0.03 | 18.32 | 0.27 | 25.33% |
| NDVI | 80.46 | -0.74 | 0.02 | 17.79 | 0.32 | 17.72 | 0.45 | 24.50% |
| B | 389.02 | 62.80 | 0.01 | 17.85 | 0.03 | 18.42 | 1.82 | 25.47% |
| 1/B | -0.18 | 79.71 | 0.01 | 17.87 | 0.03 | 18.39 | 1.67 | 25.43% |
| lnB | 8.62 | 104.49 | 0.01 | 17.86 | 0.03 | 18.40 | 1.76 | 25.44% |
| R | 89.42 | 68.62 | 0.00 | 17.94 | 0.00 | 18.56 | 1.41 | 25.67% |
| 1/R | 0.06 | 69.10 | 0.00 | 17.95 | 0.00 | 18.53 | 1.01 | 25.62% |
| lnR | 0.53 | 73.05 | 0.00 | 17.96 | 0.00 | 18.54 | 1.20 | 25.64% |
| TCARI | 9.43 | 72.53 | 0.00 | 17.91 | 0.03 | 18.72 | 1.28 | 25.89% |

**Note:** The sample sizes for the calibration and validation dataset are 136 and 68, respectively. The coefficients and intercepts are calculated based on the calibration dataset.

**Table S10** Relationships (linear model) between Jmax and published spectral indices.

| Spectral  indices | Coefficient | Intercept | Calibration | | Validation | | | |
| --- | --- | --- | --- | --- | --- | --- | --- | --- |
|  |  |  | R² | RMSE  (μmol m^-2^ s^-1^) | R² | RMSE  (μmol m^-2^ s^-1^) | Bias  (μmol m^-2^ s^-1^) | rRMSE |
| CVI | 43.47 | 43.19 | 0.38 | 43.59 | 0.53 | 39.89 | 1.25 | 21.90% |
| b | 1750.07 | -135.32 | 0.36 | 44.24 | 0.32 | 47.36 | 3.05 | 26.00% |
| GBRI | -57.34 | 368.96 | 0.35 | 44.84 | 0.32 | 47.75 | 3.29 | 26.21% |
| ExB | 466.64 | 334.51 | 0.33 | 45.37 | 0.31 | 48.43 | 4.78 | 26.59% |
| WI | -474.45 | 412.88 | 0.31 | 46.20 | 0.29 | 49.22 | 5.17 | 27.02% |
| IPCA | -2110.01 | 359.05 | 0.31 | 46.03 | 0.34 | 47.58 | -5.30 | 26.12% |
| g | -676.57 | 575.05 | 0.28 | 46.97 | 0.27 | 49.91 | 5.10 | 27.40% |
| GLI | -410.37 | 374.07 | 0.28 | 47.13 | 0.27 | 50.04 | 5.29 | 27.47% |
| ExG | -225.52 | 349.53 | 0.28 | 46.97 | 0.27 | 49.91 | 5.10 | 27.40% |
| ExGR | -134.33 | 315.20 | 0.27 | 47.58 | 0.26 | 50.49 | 5.07 | 27.72% |
| RGBVI | -359.24 | 456.97 | 0.27 | 47.52 | 0.28 | 50.33 | 5.65 | 27.63% |
| VARI | -29594.64 | 270.53 | 0.24 | 48.36 | 0.27 | 50.19 | -6.21 | 27.55% |
| GRRI | -60.40 | 332.84 | 0.23 | 48.56 | 0.20 | 51.72 | 3.81 | 28.39% |
| ExR | 325.79 | 263.06 | 0.23 | 48.60 | 0.22 | 51.51 | 4.89 | 28.28% |
| GRVI | -321.64 | 316.71 | 0.22 | 49.17 | 0.21 | 52.08 | 4.84 | 28.59% |
| SCCCI | 564.36 | -71.74 | 0.22 | 48.95 | 0.53 | 41.58 | -5.91 | 22.83% |
| MGRVI | -233.78 | 346.74 | 0.20 | 49.74 | 0.21 | 52.57 | 5.14 | 28.86% |
| GLI2 | -537.98 | 558.03 | 0.19 | 49.96 | 0.18 | 52.87 | 4.61 | 29.02% |
| MCARI | -72.79 | 275.42 | 0.18 | 50.35 | 0.04 | 56.50 | -1.68 | 31.02% |
| r | 812.18 | -10.52 | 0.17 | 50.51 | 0.15 | 53.46 | 4.19 | 29.35% |
| VIg | 14.59 | 71.14 | 0.14 | 51.55 | 0.47 | 45.82 | -4.77 | 25.15% |
| VIre | 71.68 | 12.81 | 0.14 | 51.41 | 0.47 | 45.27 | -6.51 | 24.85% |
| CIGreen | 14.59 | 85.73 | 0.14 | 51.55 | 0.47 | 45.82 | -4.77 | 25.15% |
| CIRE | 71.68 | 84.50 | 0.14 | 51.41 | 0.47 | 45.27 | -6.51 | 24.85% |
| NDRE | 408.11 | 18.53 | 0.13 | 51.71 | 0.49 | 45.33 | -6.16 | 24.89% |
| GNDVI | 516.04 | -210.92 | 0.11 | 52.26 | 0.48 | 46.29 | -4.42 | 25.41% |
| 1/G | 7.82 | 65.33 | 0.10 | 52.79 | 0.17 | 53.50 | -5.74 | 29.37% |
| B | 3276.72 | 113.66 | 0.09 | 52.89 | 0.06 | 55.64 | 3.67 | 30.55% |
| lnG | -114.07 | -124.98 | 0.09 | 53.03 | 0.17 | 53.76 | -6.08 | 29.51% |
| NDI | 309.96 | 15.06 | 0.09 | 52.99 | 0.46 | 47.71 | -5.69 | 26.19% |
| MCARI2 | 168.23 | 143.24 | 0.09 | 52.93 | 0.43 | 47.51 | -4.59 | 26.08% |
| LCI | 309.96 | 15.06 | 0.09 | 52.99 | 0.46 | 47.71 | -5.69 | 26.19% |
| G | -1594.20 | 291.32 | 0.08 | 53.30 | 0.16 | 54.07 | -6.25 | 29.68% |
| lnB | 72.21 | 463.18 | 0.08 | 53.21 | 0.06 | 55.72 | 3.08 | 30.59% |
| TCARI | 119.00 | 201.41 | 0.08 | 53.14 | 0.00 | 57.98 | -0.39 | 31.83% |
| 1/B | -1.44 | 254.26 | 0.07 | 53.59 | 0.05 | 55.88 | 2.27 | 30.68% |
| R | 1530.80 | 140.67 | 0.05 | 54.14 | 0.01 | 56.90 | 2.34 | 31.24% |
| RE | -574.76 | 307.62 | 0.05 | 54.04 | 0.00 | 57.86 | -6.62 | 31.76% |
| 1/RE | 26.92 | 57.54 | 0.05 | 53.97 | 0.01 | 57.90 | -6.61 | 31.79% |
| lnRE | -125.27 | -9.15 | 0.05 | 54.01 | 0.01 | 57.88 | -6.63 | 31.77% |
| CI2 | 47.32 | 78.93 | 0.05 | 54.01 | 0.25 | 52.63 | -1.66 | 28.89% |
| lnR | 45.09 | 346.16 | 0.04 | 54.43 | 0.01 | 57.03 | 1.81 | 31.31% |
| 1/R | -1.12 | 226.00 | 0.03 | 54.76 | 0.00 | 57.15 | 0.98 | 31.37% |
| 1/NIR | -32.63 | 249.10 | 0.03 | 54.75 | 0.29 | 52.40 | -1.03 | 28.77% |
| RBRI | -82.90 | 293.25 | 0.03 | 54.70 | 0.12 | 55.24 | -3.63 | 30.33% |
| IKAW | -233.56 | 215.54 | 0.03 | 54.62 | 0.12 | 55.18 | -3.53 | 30.29% |
| NIR | 96.89 | 134.50 | 0.02 | 55.06 | 0.27 | 53.67 | -1.49 | 29.46% |
| lnNIR | 57.88 | 223.42 | 0.02 | 54.91 | 0.28 | 52.99 | -1.27 | 29.09% |
| NDVI | -247.15 | 404.98 | 0.02 | 54.99 | 0.13 | 58.95 | 0.19 | 32.36% |
| lnre | -0.19 | 240.07 | 0.01 | 55.25 | 0.13 | 58.64 | -0.59 | 32.19% |
| MSAVI2 | 54.94 | 142.09 | 0.01 | 55.36 | 0.28 | 55.21 | -2.18 | 30.31% |
| MSR | 133.19 | 223.97 | 0.01 | 55.11 | 0.27 | 53.79 | -1.63 | 29.53% |
| SAVI | 72.94 | 133.52 | 0.01 | 55.35 | 0.28 | 55.08 | -2.06 | 30.24% |
| DVI | 77.05 | 146.85 | 0.01 | 55.22 | 0.26 | 54.37 | -1.80 | 29.85% |
| EVI | 72.09 | 127.07 | 0.01 | 55.18 | 0.29 | 54.08 | -1.99 | 29.69% |
| INT | 244.64 | 174.57 | 0.00 | 55.48 | 0.02 | 57.36 | -1.38 | 31.49% |
| VIr | -0.62 | 196.05 | 0.00 | 55.42 | 0.12 | 58.12 | -1.30 | 31.91% |
| MSAVI | -25.57 | 205.29 | 0.00 | 55.49 | 0.25 | 57.66 | -1.82 | 31.65% |
| OSAVI | 21.48 | 166.99 | 0.00 | 55.49 | 0.27 | 56.81 | -2.07 | 31.19% |
| MTVI2 | -42.95 | 218.70 | 0.00 | 55.39 | 0.19 | 58.64 | -1.60 | 32.19% |
| SIPI | 111.50 | 70.99 | 0.00 | 55.49 | 0.20 | 57.45 | -1.72 | 31.54% |
| RVI | -0.62 | 196.05 | 0.00 | 55.42 | 0.12 | 58.12 | -1.30 | 31.91% |
| TVI | 0.72 | 162.10 | 0.00 | 55.41 | 0.24 | 55.66 | -1.88 | 30.56% |

**Note:** The sample sizes for the calibration and validation dataset are 136 and 68, respectively. The coefficients and intercepts are calculated based on the calibration dataset.

**Table S11** Modeling results of PLSR model with or without LASSO variable selection for estimating the photosynthetic traits of tea chrysanthemum.

| Variable selection | Photosynthetic  parameters | Calibration | | Validation | | | | Hyperparameters |
| --- | --- | --- | --- | --- | --- | --- | --- | --- |
|  |  | R² | RMSE (μmol m^-2^ s^-1^) | R² | RMSE (μmol m^-2^ s^-1^) | Bias (μmol m^-2^ s^-1^) | rRMSE |  |
| With LASSO | Vcmax | 0.70 | 10.18 | 0.70 | 10.18 | 0.75 | 14.08% | ncomponents = 7 |
|  | Jmax | 0.61 | 29.74 | 0.63 | 35.08 | 3.20 | 19.26% | ncomponents = 11 |
| Without LASSO | Vcmax | 0.70 | 9.76 | 0.68 | 10.38 | -0.34 | 14.36% | ncomponents = 7 |
|  | Jmax | 0.51 | 38.80 | 0.59 | 36.91 | -1.43 | 20.26% | ncomponents = 3 |

**Note:** Vcmax is the maximum carboxylation rate, Jmax is the maximum electron transfer rate, PLSR is partial least squares regression, with a calibration dataset of 136 and a validation dataset of 68, the hyperparameters are determined by 10-fold cross validation. After applying LASSO, the number of input variables for estimating Vcmax and Jmax was reduced to 9 and 18, respectively, compared to 63 input variables used without LASSO.


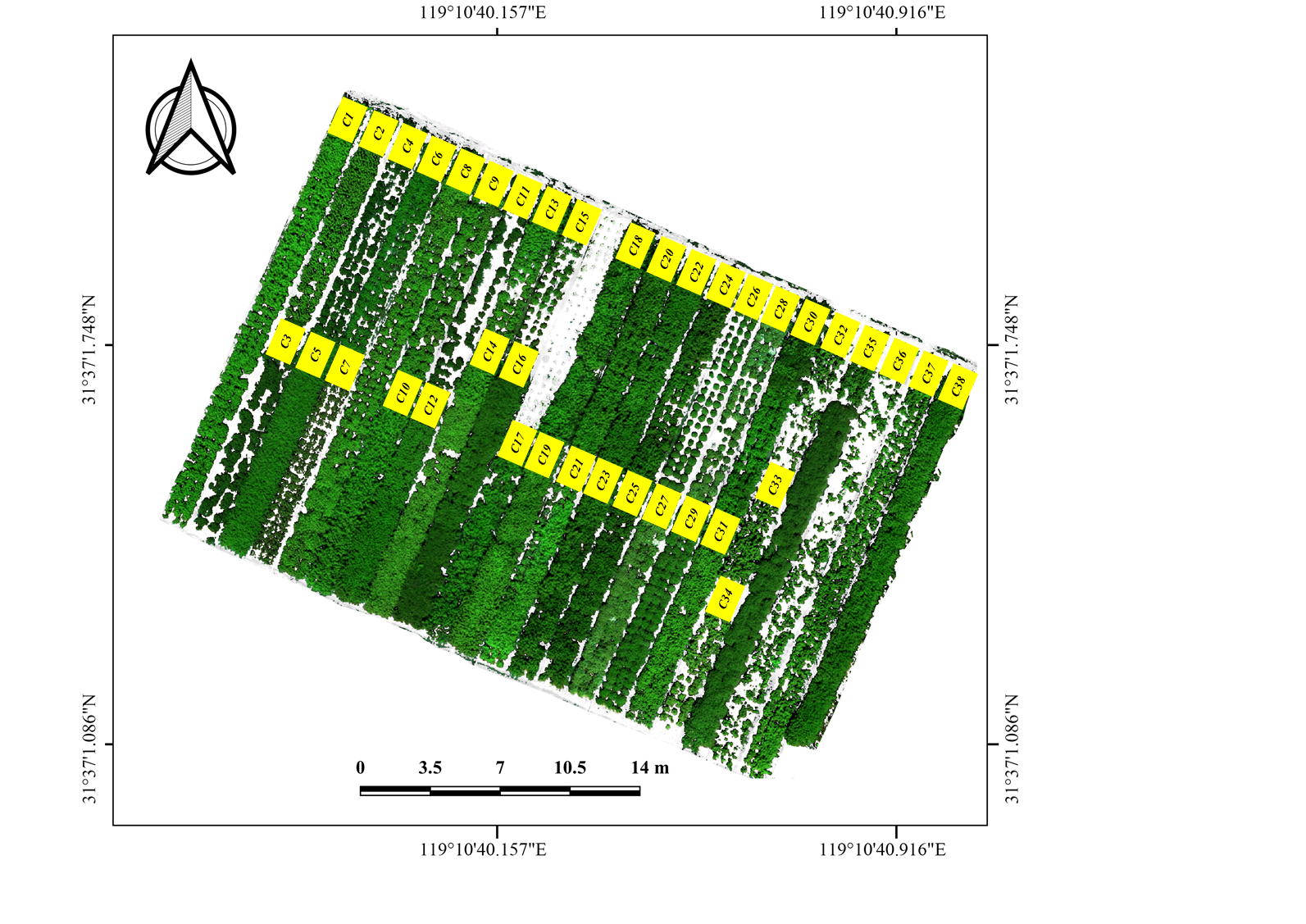


**Fig.S1.** Tea chrysanthemum planting map in the experiment 6. The imagery was captured on 19 September 2023.


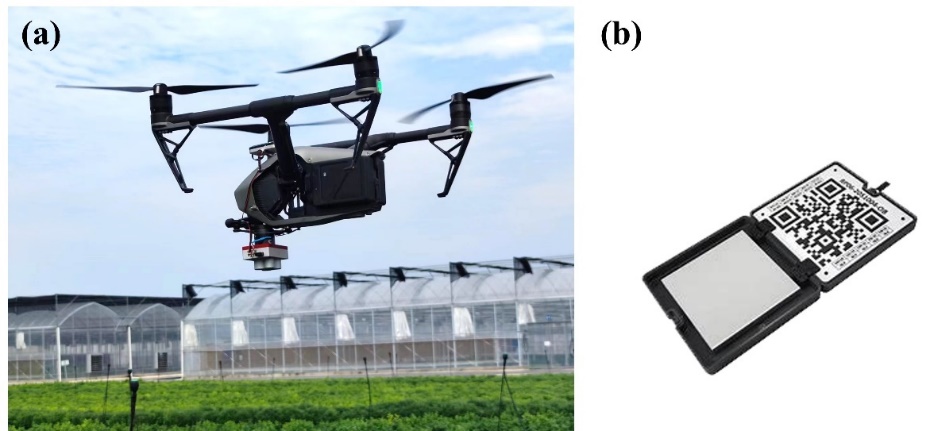


**Fig.S2.** MicaSense multispectral camera RedEdge-MX mounted on the DJI Inspire 2 UAV (a) and calibration reference panel (b).


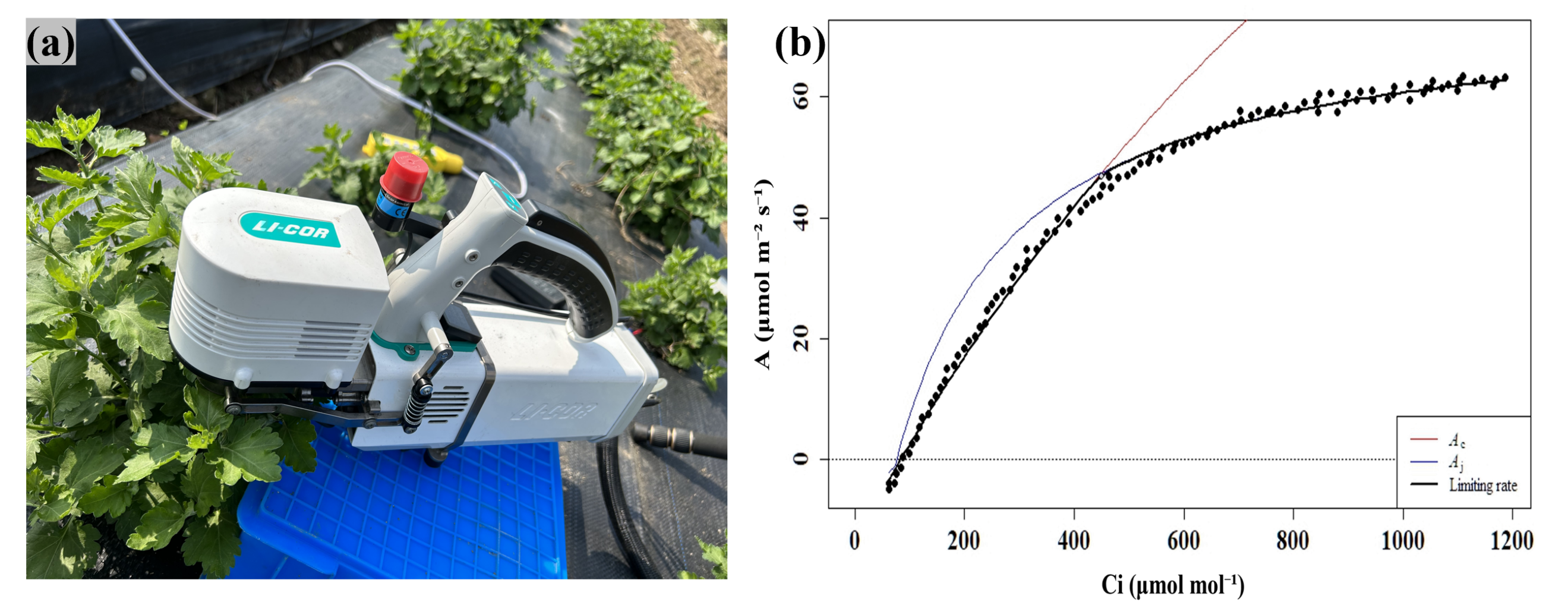


**Fig.S3.** Measurement of the A-Ci response curve (a) and fitting of the A-Ci curve (b). A denotes the net photosynthetic rate and Ci represents the intercellular CO_2_ concentration. The symbols correspond to the measurements, whereas the black line depicts the fitted FvCB model of photosynthesis. Colored lines indicate the two photosynthetic rates in the FvCB model.


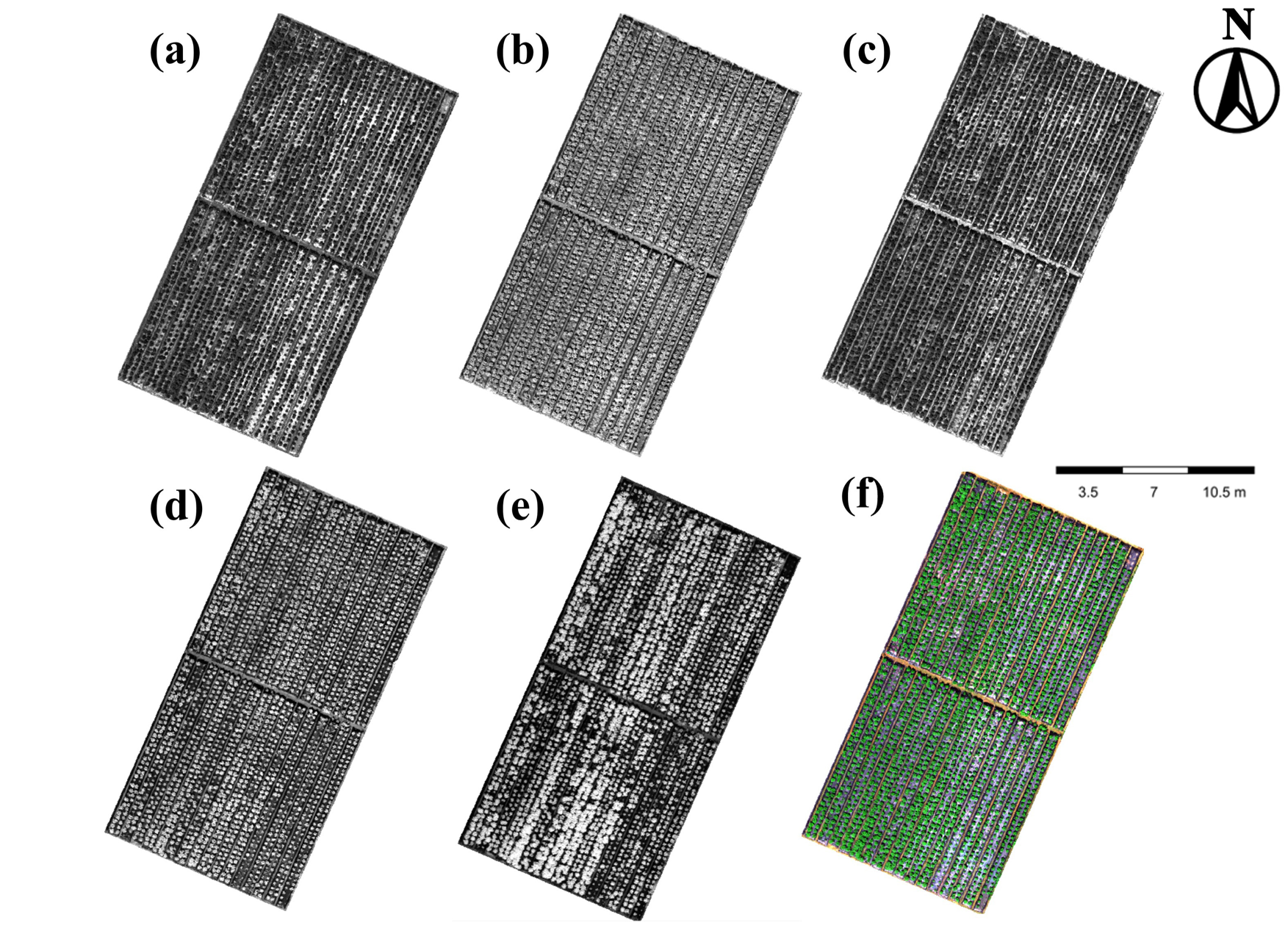


**Fig.S4.** Multispectral images: blue band (a), green band (b), red band (c), red edge band (d), near-infrared band (e), and true color image (f). The imagery used in this study was captured on August 13, 2023, in Lishui.


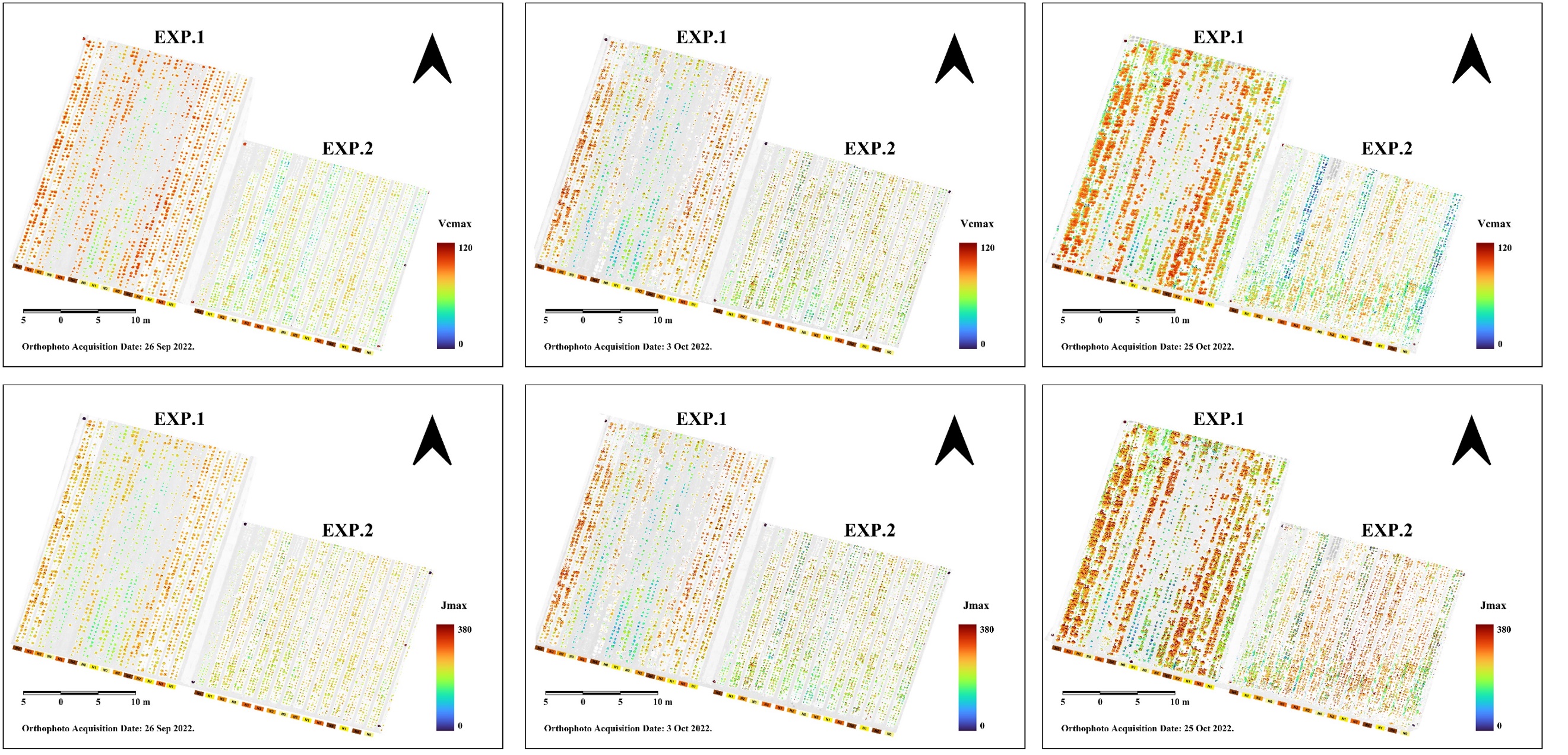


**Fig.S5.** Photosynthetic trait maps generated using established PLSR models for Experiments 1 and 2 across different UAV flight dates.


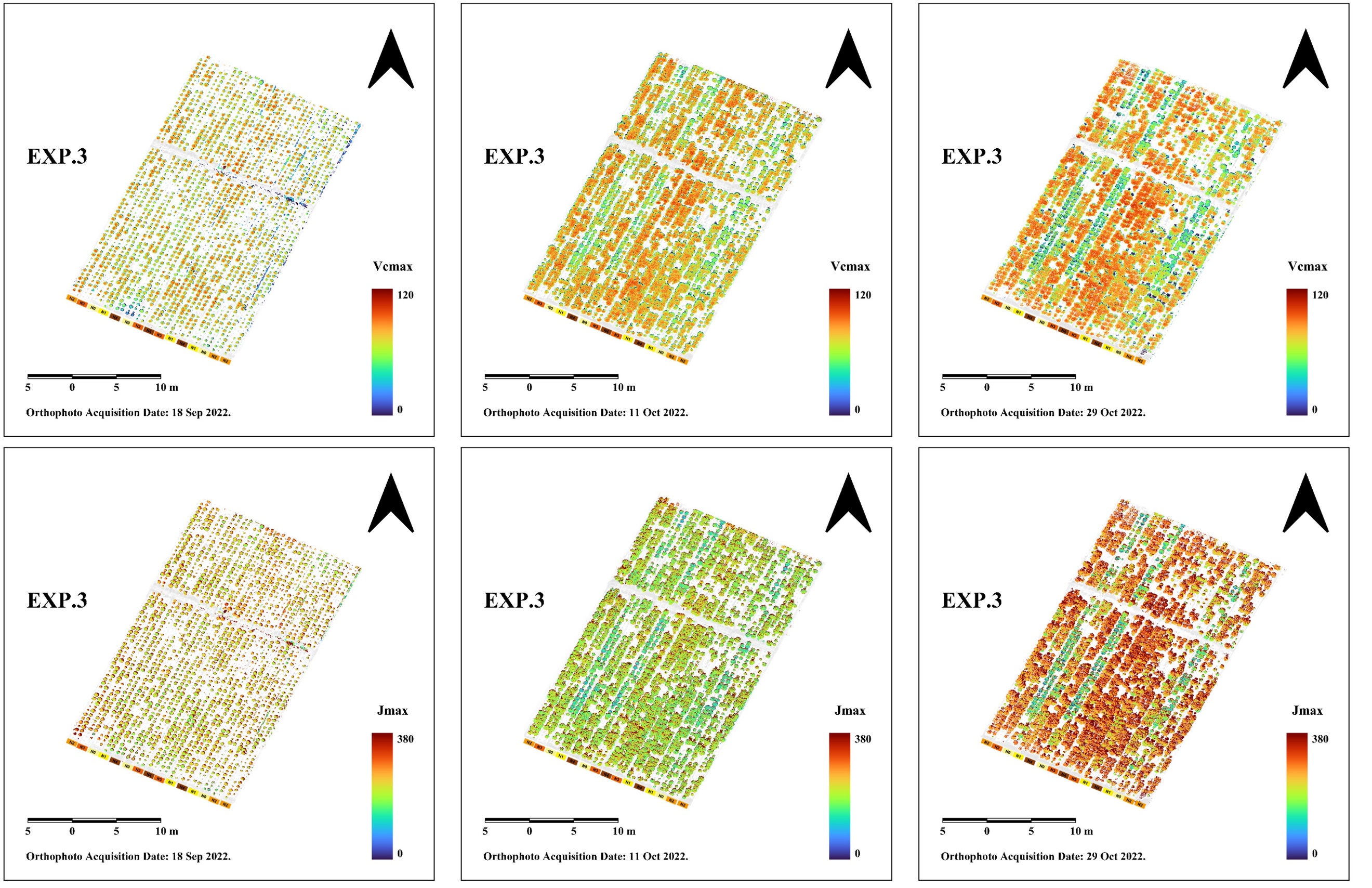


**Fig.S6.** Photosynthetic trait maps generated using established PLSR models for Experiment 3 across different UAV flight dates.


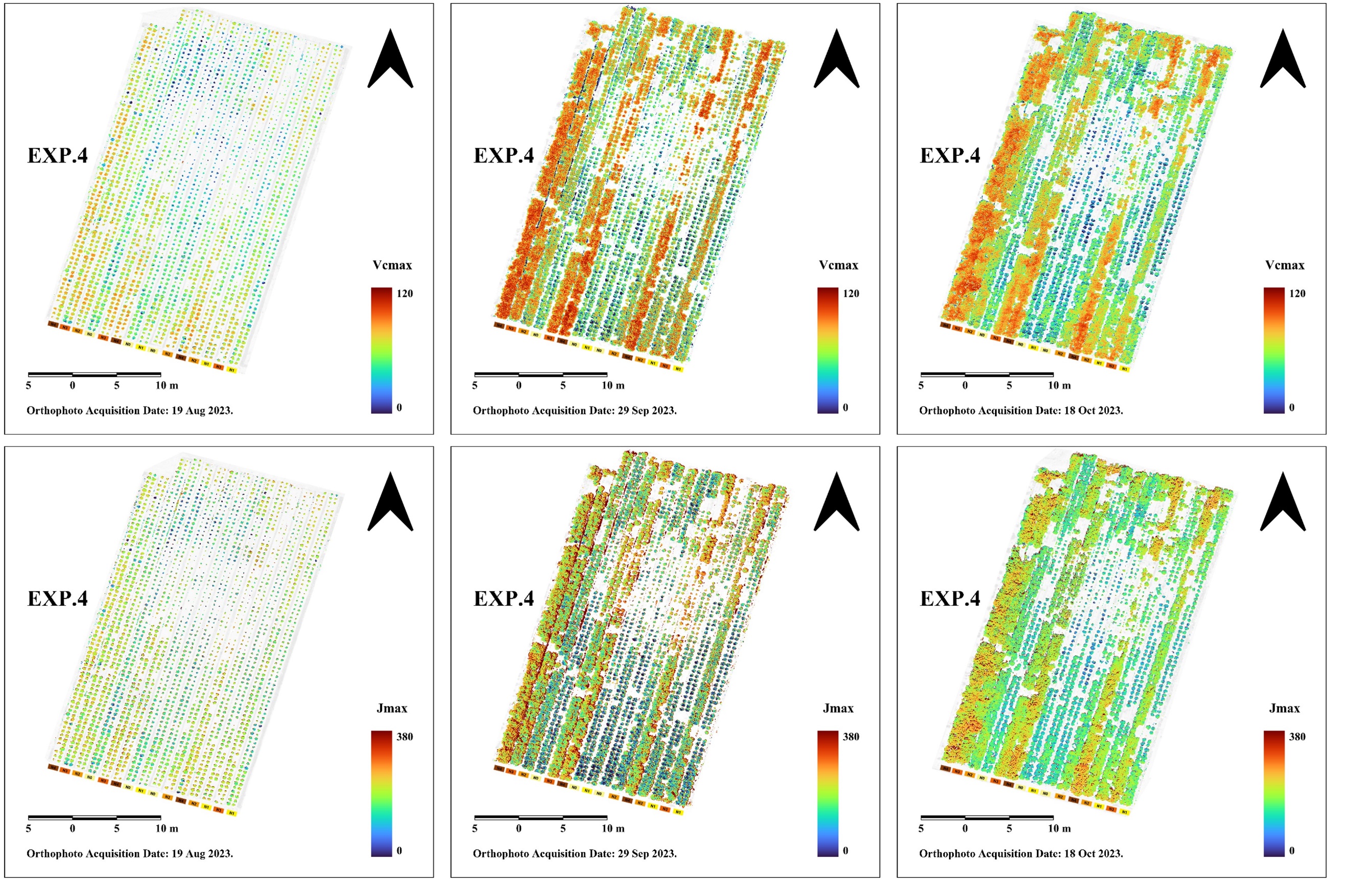


**Fig.S7.** Photosynthetic trait maps generated using established PLSR models for Experiment 4 across different UAV flight dates.


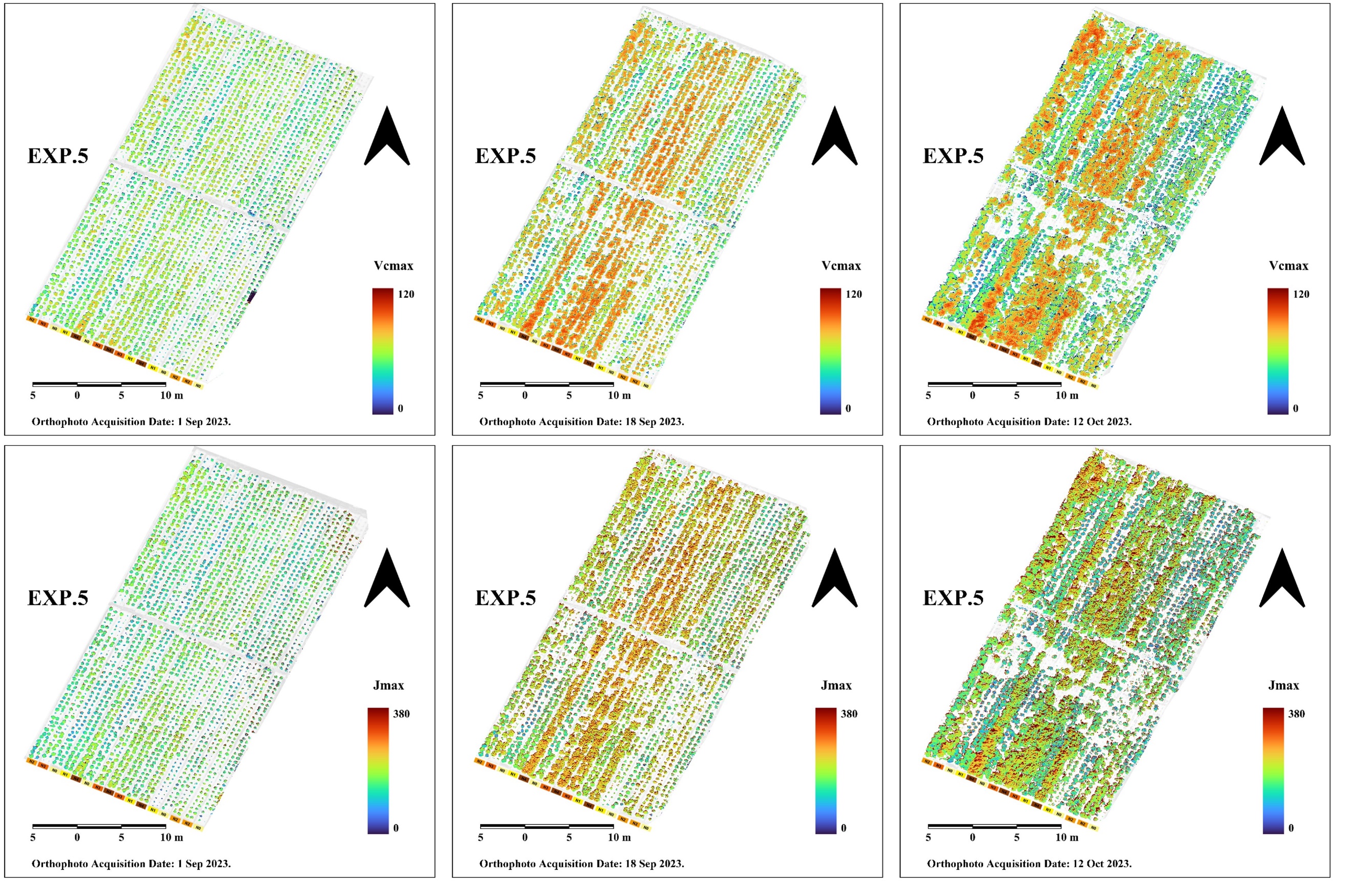


**Fig.S8.** Photosynthetic trait maps generated using established PLSR models for Experiment 5 across different UAV flight dates.


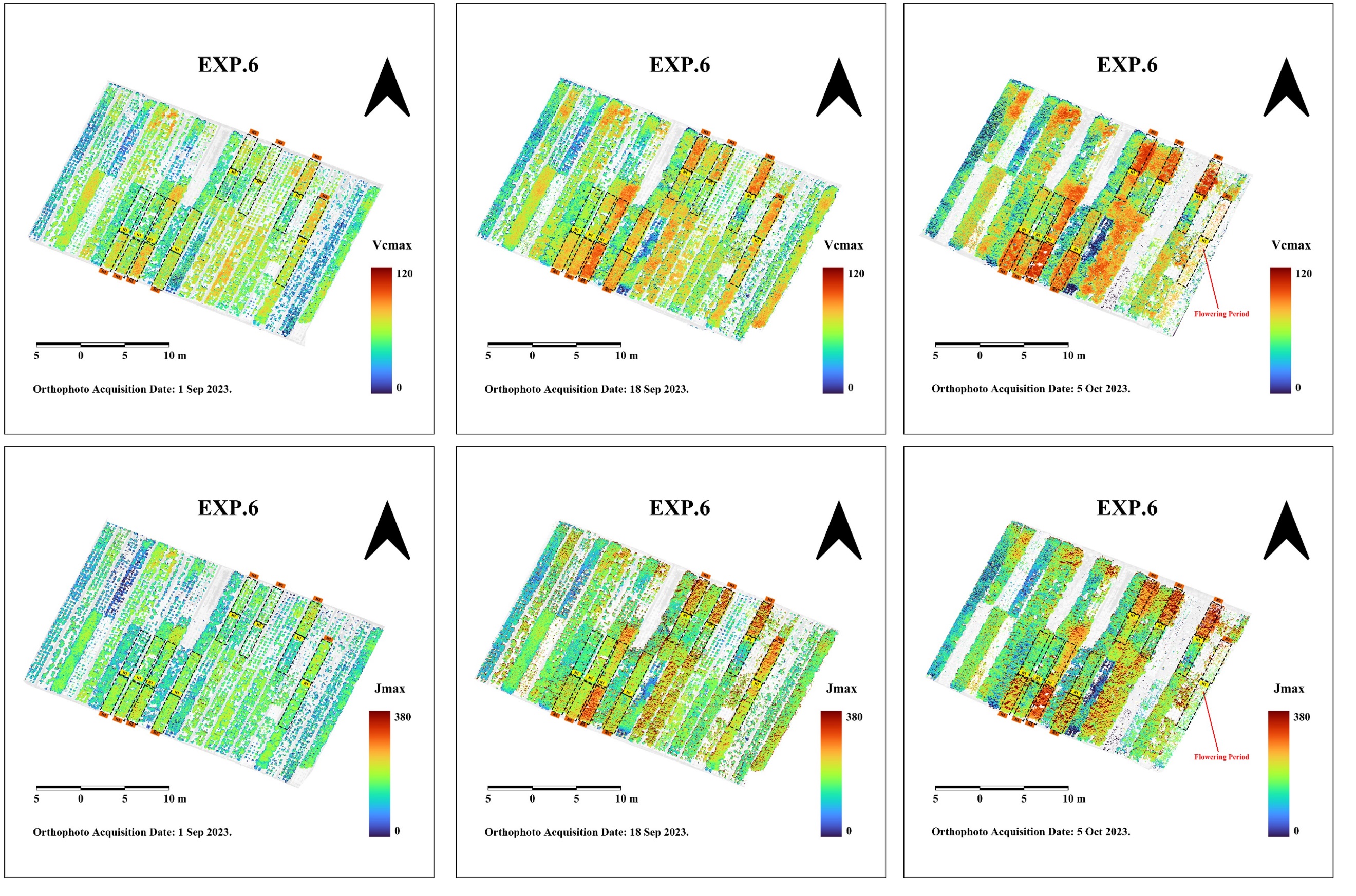


**Fig.S9.** Photosynthetic trait maps generated using established PLSR models for Experiment 6 across different UAV flight dates.


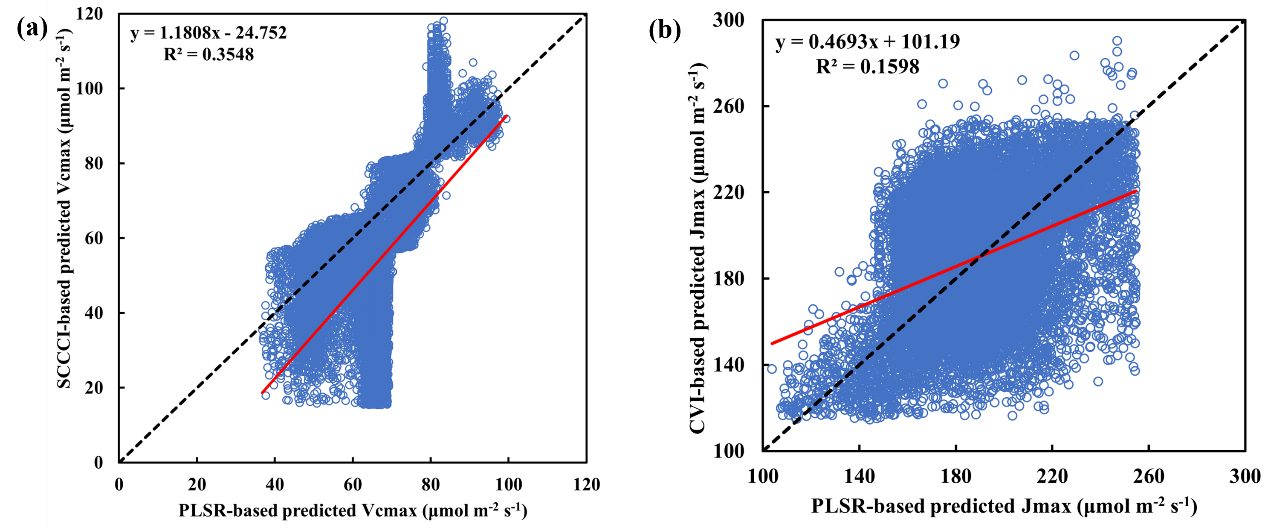


**Fig.S10.** Correlation between the photosynthetic trait predicted values of all pixels in the spatial mapping generated by spectral index and PLSR prediction models (Fig. 9). (a) Vcmax predicted by SCCCI and PLSR models, (b) Jmax predicted by CVI and PLSR models.


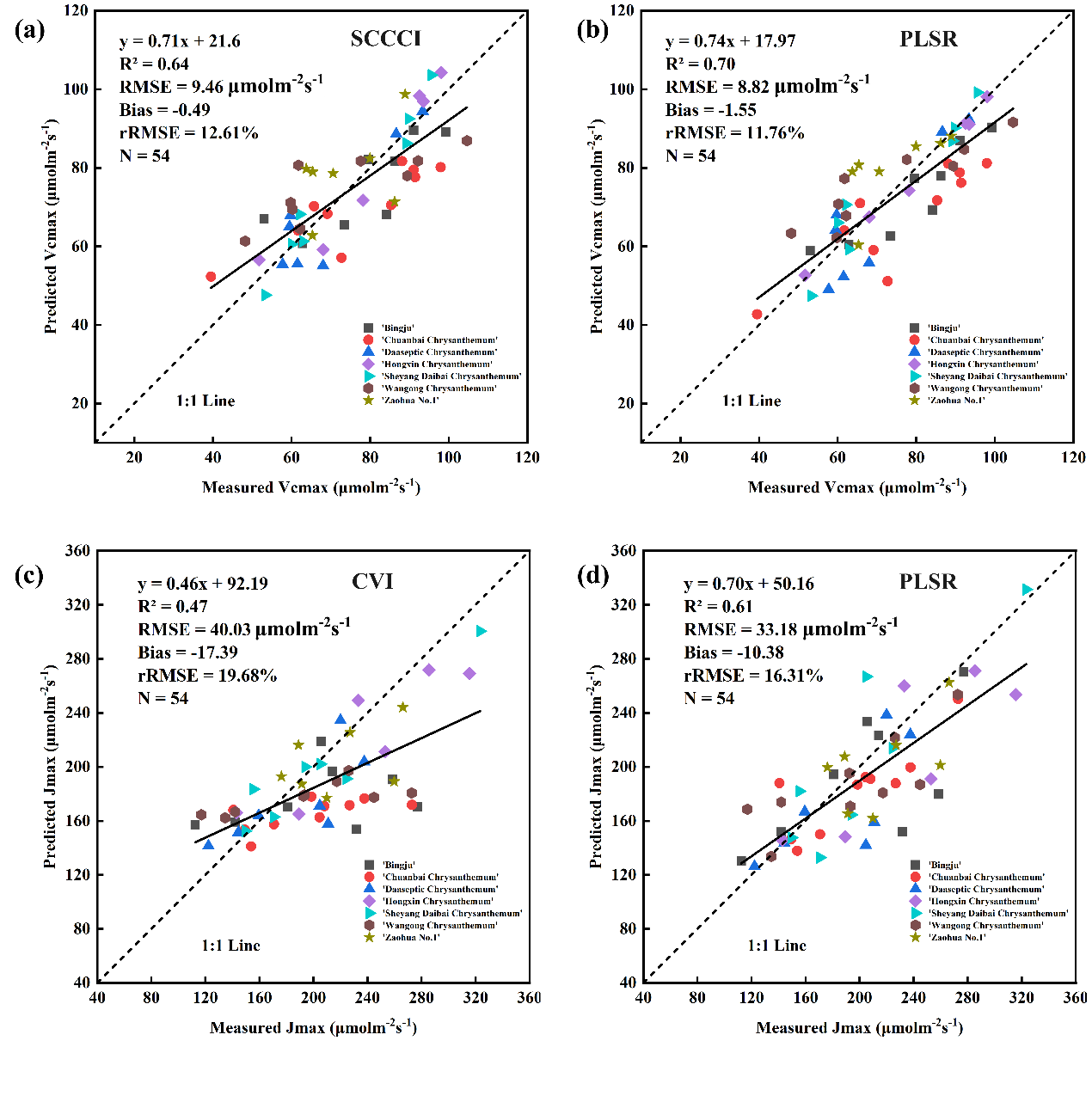


**Fig.S11.** Relationship between the measured and predicted Vcmax and Jmax of tea chrysanthemum using optimal spectral indices and the PLSR model, based on the data from experiment 6. (a) SCCCI for Vcmax, (b) PLSR for Vcmax, (c) CVI for Jmax, and (d) PLSR for Jmax.


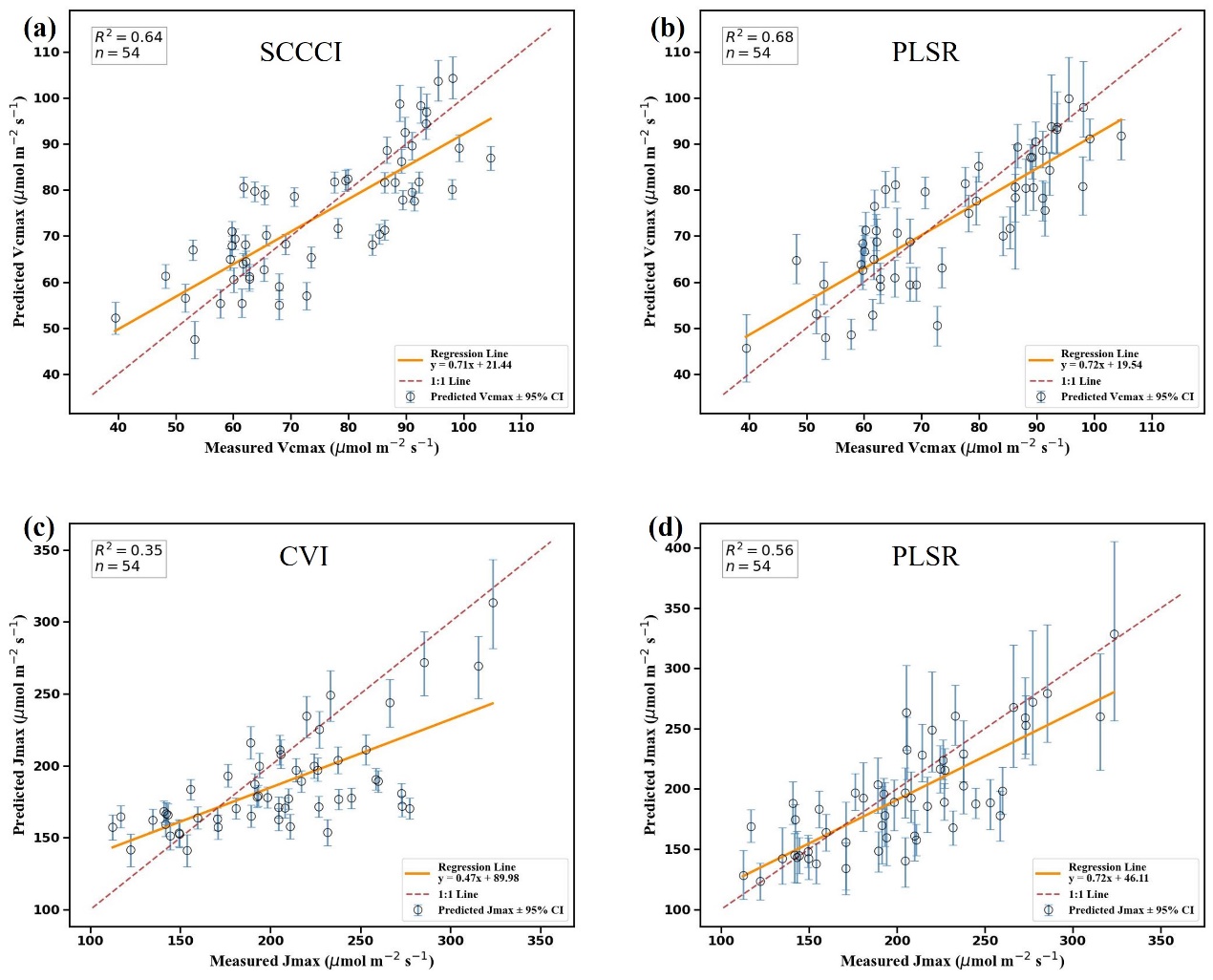


**Fig.S12.** Relationship between measured and predicted Vcmax and Jmax, along with prediction uncertainty, using the bootstrapping method (B = 1000) for tea chrysanthemum in experiment 6. Predictions were made using optimal spectral indices and the PLSR model: (a) SCCCI for Vcmax, (b) PLSR for Vcmax, (c) CVI for Jmax, and (d) PLSR for Jmax.


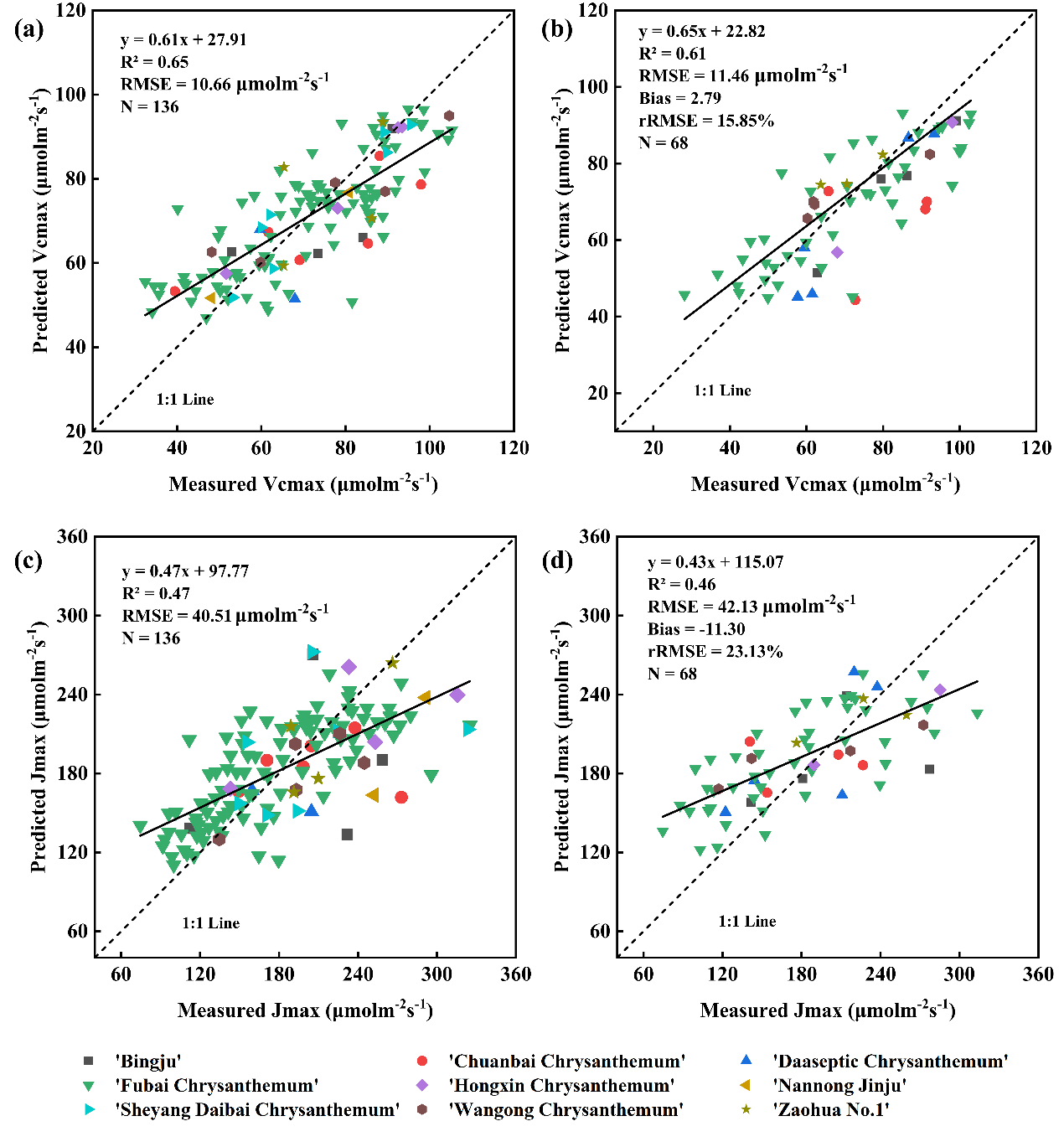


**Fig.S13.** The relationship between the measured and predicted photosynthetic parameters of tea chrysanthemum using an optimized random forest (RF) model: (a) Vcmax in calibration, (b) Vcmax in validation, (c) Jmax in calibration, and (d) Jmax in validation. The model was trained with 10-fold cross-validation to select optimal hyperparameters (n_estimators = 1000, max_features = max_features_opt) and implemented via the “scikit-learn 1.5.1” package in Python.


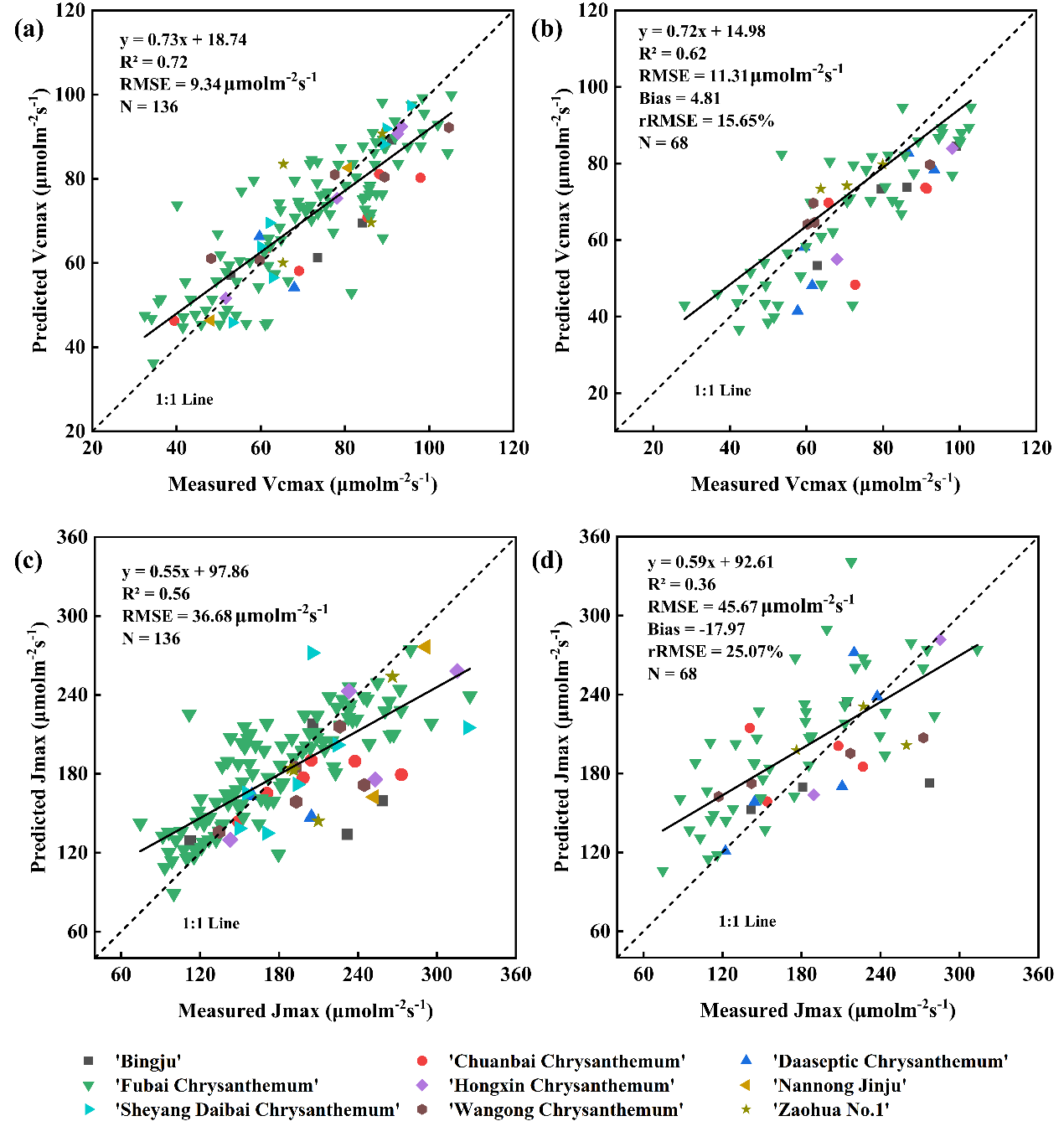


**Fig.S14.** The relationship between the measured and predicted photosynthetic parameters of tea chrysanthemum using an optimized support vector machine (SVM) regression model: (a) Vcmax in calibration, (b) Vcmax in validation, (c) Jmax in calibration, and (d) Jmax in validation. The model was trained with 10-fold cross-validation to select optimal hyperparameters (kernel = rbf, C = C_opt, gamma = gamma_opt) and implemented via the “scikit-learn 1.5.1” package in Python.


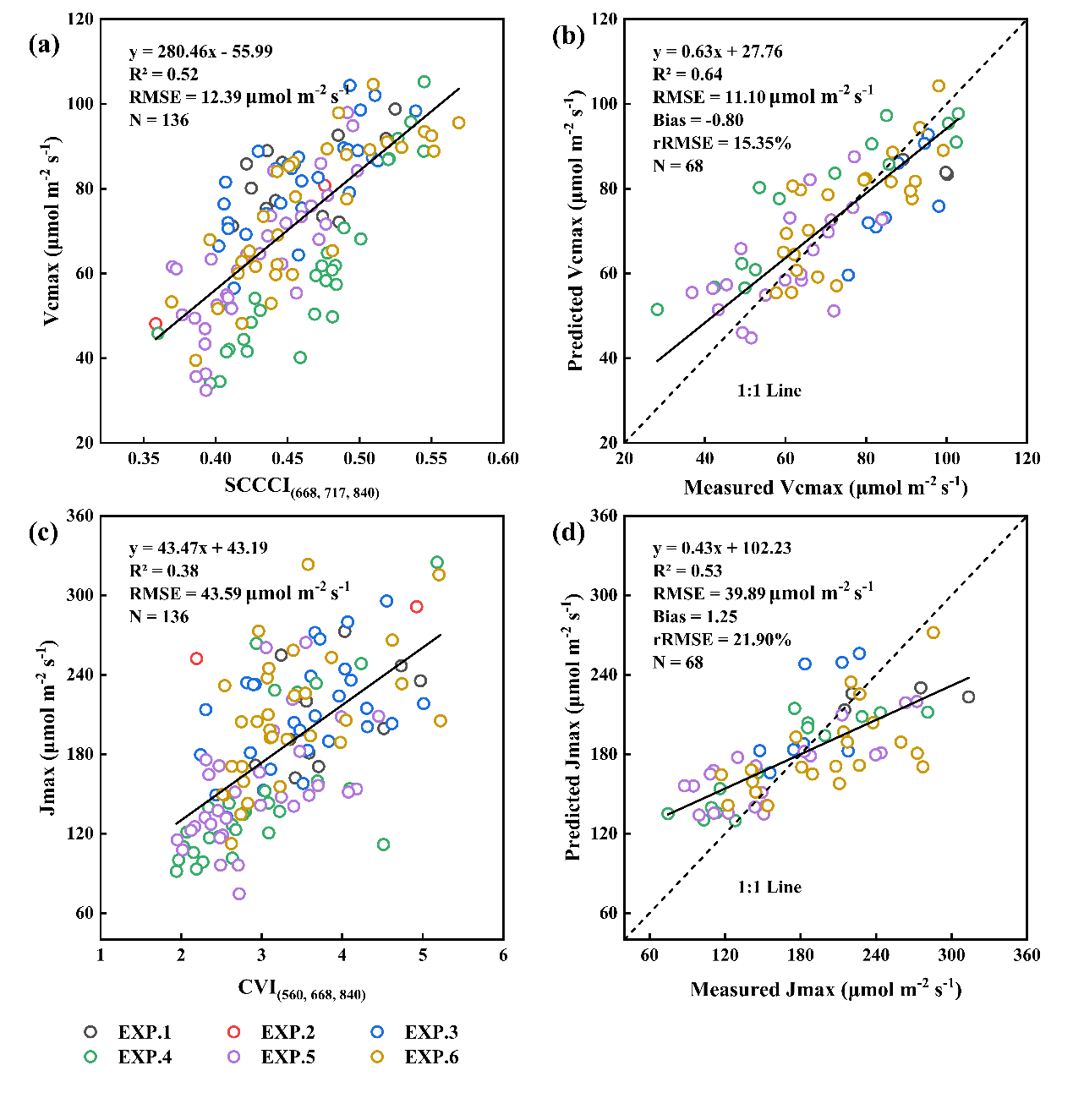


**Fig.S15.** Relationships between photosynthetic traits and optimal spectral indices with different experiments: (a) SCCCI for Vcmax, (c) CVI for Jmax. Validation results for the estimation of photosynthetic traits using SCCCI (b) and CVI (d).


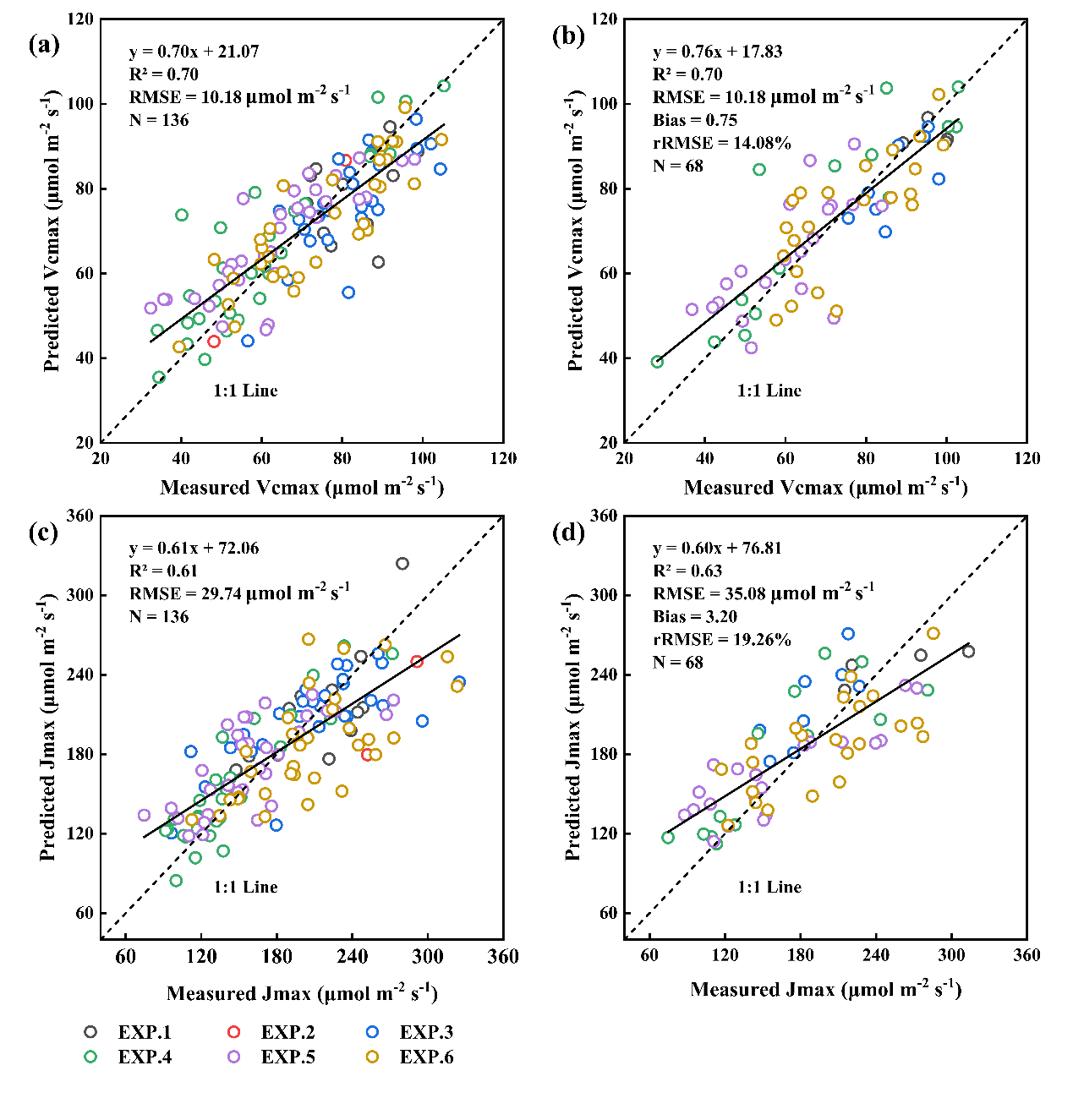


**Fig.S16.** Relationship between the measured and predicted Vcmax and Jmax of tea chrysanthemum using PLSR model with different experiments: (a) PLSR estimation of Vcmax in the calibration dataset, (b) PLSR estimation of Vcmax in the validation dataset, (c) PLSR estimation of Jmax in the calibration dataset, (d) PLSR estimation of Jmax in the validation dataset.

**Reference:**

[1] Liu Y, Wu C, Sonnentag O, et al. Using the red chromatic coordinate to characterize the phenology of forest canopy photosynthesis. Agricultural and Forest Meteorology 2020;285-286.

[2] Liu S, Jin X, Nie C, et al. Estimating leaf area index using unmanned aerial vehicle data: shallow vs. deep machine learning algorithms. Plant Physiology 2021;187:1551-1576.

[3] Ahmad IS, , Reid JF. Evaluation of colour representations for maize images. Journal of Agricultural Engineering Research 1996;63:185-195.

[4] Tucker CJ. Red and photographic infrared linear combinations for monitoring vegetation. Remote Sensing of Environment 1979;8:127-150.

[5] Woebbecke D, Meyer G, Von Bargen K, et al. Plant species identification, size, and enumeration using machine vision techniques on near-binary images. Vol 1836: SPIE. 1993.

[6] Woebbecke DM, E. Meyer G, Von Bargen K, et al. Color indices for weed identification under various soil, residue, and lighting conditions. Transactions of the ASAE 1995;38:259-269.

[7] Kawashima S, Nakatani M. An algorithm for estimating chlorophyll content in leaves using a video camera. Annals of Botany 1998;81:49-54.

[8] Louhaichi M, Borman MM, Johnson DE. Spatially located platform and aerial photography for documentation of grazing impacts on wheat. Geocarto International 2001;16:65-70.

[9] Gitelson AA, Kaufman YJ, Stark R, et al. Novel algorithms for remote estimation of vegetation fraction. Remote Sensing of Environment 2002;80:76-87.

[10] Mao W, Wang Y, Wang Y. Real-time detection of between-row weeds using machine vision. In. St. Joseph, MI. 2003.

[11] Saberioon MM, Amin MSM, Anuar AR, et al. Assessment of rice leaf chlorophyll content using visible bands at different growth stages at both the leaf and canopy scale. International Journal of Applied Earth Observation and Geoinformation 2014;32:35-45.

[12] Gamon JA, Surfus JS. Assessing leaf pigment content and activity with a reflectometer. New Phytologist 2002;143:105-117.

[13] Bendig J, Yu K, Aasen H, et al. Combining UAV-based plant height from crop surface models, visible, and near infrared vegetation indices for biomass monitoring in barley. International Journal of Applied Earth Observation and Geoinformation 2015;39:79-87.

[14] Gitelson AA, Merzlyak MN. Remote estimation of chlorophyll content in higher plant leaves. International Journal of Remote Sensing 1997;18:2691-2697.

[15] Ramoelo A, Skidmore AK, Cho MA, et al. Regional estimation of savanna grass nitrogen using the red-edge band of the spaceborne RapidEye sensor. International Journal of Applied Earth Observation and Geoinformation 2012;19:151-162.

[16] Portz G, Molin JP, Jasper J. Active crop sensor to detect variability of nitrogen supply and biomass on sugarcane fields. Precision Agriculture 2011;13:33-44.

[17] Qi J, Chehbouni A, Huete AR, et al. A modified soil adjusted vegetation index. Remote Sensing of Environment 1994;48:119-126.

[18] Rondeaux G, Steven M, Baret F. Optimization of soil-adjusted vegetation indices. Remote Sensing of Environment 1996;55:95-107.

[19] Chen JM. Evaluation of vegetation indices and a modified simple ratio for boreal applications. Canadian Journal of Remote Sensing 1996;22:229-242.

[20] Haboudane D, Miller JR, Pattey E, et al. Hyperspectral vegetation indices and novel algorithms for predicting green LAI of crop canopies: Modeling and validation in the context of precision agriculture. Remote Sensing of Environment 2004;90:337-352.

[21] Huete AR. A soil-adjusted vegetation index (SAVI). Remote Sensing of Environment 1988;25:295-309.

[22] Fitzgerald G, Rodriguez D, O’Leary G. Measuring and predicting canopy nitrogen nutrition in wheat using a spectral index—The canopy chlorophyll content index (CCCI). Field Crops Research 2010;116:318-324.

[23] Rouse JW, Haas RH, Schell JA, et al. Monitoring vegetation systems in the great plains with ERTS. In: 3rd Earth Resources Technology Satellite-1 Symposium, NASA SP-351, Greenbelt, MD, pp. 301–317. 1974.

[24] Raper TB, Varco JJ. Canopy-scale wavelength and vegetative index sensitivities to cotton growth parameters and nitrogen status. Precision Agriculture 2014;16:62-76.

[25] Daughtry CST, Walthall CL, Kim MS, et al. Estimating corn Leaf chlorophyll concentration from leaf and canopy reflectance. Remote Sensing of Environment 2000;74:229-239.

[26] Haboudane D, Miller JR, Tremblay N, et al. Integrated narrow-band vegetation indices for prediction of crop chlorophyll content for application to precision agriculture. Remote Sensing of Environment 2002;81:416-426.

[27] Gitelson AA, Merzlyak MN. Signature Analysis of Leaf Reflectance Spectra: Algorithm Development for Remote Sensing of Chlorophyll. Journal of Plant Physiology 1996;148:494-500.

[28] Penuelas J, Baret F, Filella I. Semi empirical-indices to assess carotenoids/chlorophyll A ratio from leaf spectral reflectance. In: 6. Symposium International de l'ISPRS. Val d'Isere, France, Toulouse (france). 1994.

[29] Gitelson AA, Viña A, Ciganda V, et al. Remote estimation of canopy chlorophyll content in crops. Geophysical Research Letters 2005;32.

[30] Jordan CF. Derivation of leaf-area index from quality of light on the forest floor. 1969;50:663-666.

[31] Person R. Remote mapping of standing crop biomass for estimation of the productivity of the short-grass Prairie, Pawnee National Grasslands, Colorado. 1972.

[32] Daughtry CST, Gallo KP, Goward SN, et al. Spectral estimates of absorbed radiation and phytomass production in corn and soybean canopies. Remote Sensing of Environment 1992;39:141-152.

[33] Vincini M, Frazzi E, D’Alessio P. A broad-band leaf chlorophyll vegetation index at the canopy scale. Precision Agriculture 2008;9:303-319.

[34] Datt B. A new reflectance index for remote sensing of chlorophyll content in higher plants: Tests using eucalyptus leaves. Journal of Plant Physiology 1999;154:30-36.

[35] Huete A, Didan K, Miura T, et al. Overview of the radiometric and biophysical performance of the MODIS vegetation indices. Remote Sensing of Environment 2002;83:195-213.

[36] Broge NH, Leblanc E. Comparing prediction power and stability of broadband and hyperspectral vegetation indices for estimation of green leaf area index and canopy chlorophyll density. Remote Sensing of Environment 2001;76:156-172.
